# *Plasmodium berghei* NK65 in Combination with IFN-γ Induces Endothelial Glucocorticoid Resistance *via* Sustained Activation of p38 and JNK

**DOI:** 10.3389/fimmu.2017.01199

**Published:** 2017-09-28

**Authors:** Karolina A. Zielińska, Lode de Cauwer, Sofie Knoops, Kristof Van der Molen, Alexander Sneyers, Jonathan Thommis, J. Brian De Souza, Ghislain Opdenakker, Karolien De Bosscher, Philippe E. Van den Steen

**Affiliations:** ^1^Laboratory of Immunobiology, Department of Microbiology and Immunology, Rega Institute for Medical Research, KU Leuven, Leuven, Belgium; ^2^Receptor Research Laboratories, Nuclear Receptor Lab, VIB-UGent Center for Medical Biotechnology, Ghent, Belgium; ^3^Faculty of Infectious and Tropical Diseases, Department of Immunology and Infection, London School of Hygiene and Tropical Medicine, London, United Kingdom

**Keywords:** malaria, glucocorticoids, endothelium, MAPK, interferon-γ, dexamethasone

## Abstract

Malaria-associated acute respiratory distress syndrome (MA-ARDS) is an often lethal complication of malaria. Currently, no adequate therapy for this syndrome exists. Although glucocorticoids (GCs) have been used to improve clinical outcome of ARDS, their therapeutic benefits remain unclear. We previously developed a mouse model of MA-ARDS, in which dexamethasone treatment revealed GC resistance. In the present study, we investigated GC sensitivity of mouse microvascular lung endothelial cells stimulated with interferon-γ (IFN-γ) and *Plasmodium berghei* NK65 (*Pb*NK65). Upon challenge with IFN-γ alone, dexamethasone inhibited the expression of CCL5 (RANTES) by 90% and both CCL2 (MCP-1) and CXCL10 (IP-10) by 50%. Accordingly, whole transcriptome analysis revealed that dexamethasone differentially affected several gene clusters and in particular inhibited a large cluster of IFN-γ-induced genes, including chemokines. In contrast, combined stimulation with IFN-γ and *Pb*NK65 extract impaired inhibitory actions of GCs on chemokine release, without affecting the capacity of the GC receptor to accumulate in the nucleus. Subsequently, we investigated the effects of GCs on two signaling pathways activated by IFN-γ. Dexamethasone left phosphorylation and protein levels of signal transducer and activator of transcription 1 (STAT1) unhampered. In contrast, dexamethasone inhibited the IFN-γ-induced activation of two mitogen-activated protein kinases (MAPK), JNK, and p38. However, *Pb*NK65 extract abolished the inhibitory effects of GCs on MAPK signaling, inducing GC resistance. These data provide novel insights into the mechanisms of GC actions in endothelial cells and show how malaria may impair the beneficial effects of GCs.

## 1 Introduction

Malaria remains a life-threatening disease with negative impact on social and political stability. More than 200 million clinical cases and around 400,000 deaths are reported annually (WHO website). Most malaria infections are uncomplicated but various complications including cerebral malaria (CM), severe malarial anemia, and malaria-associated acute respiratory distress syndrome (MA-ARDS) cause the majority of deaths (1, 2). MA-ARDS, which occurs in adults, constitutes an important but insufficiently studied complication of malaria (3, 4). MA-ARDS is characterized by lung edema and impaired gas exchange (2). Parasite-infected erythrocytes adhere to the endothelium (parasite sequestration) in the lungs and this triggers leukocyte infiltration and proinflammatory cytokine production (2, 3). Endothelial cells are activated by these cytokines and are likely the first cells altered in the lungs during ARDS (5). Also, parasite products such as hemozoin and histidine-rich protein II have been shown to activate endothelium and increase endothelial barrier permeability (6, 7). Increased permeability of the microvascular barrier is typical in acute inflammation and plays a central role in the pathogenesis of ARDS (5, 8, 9). Moreover, this increased permeability results in interstitial edema and facilitates leukocyte infiltration. Immune cells play a crucial role in the development of MA-ARDS. Abundant monocyte and macrophage infiltrates both inside the blood capillaries and in the interstitium are found in postmortem histological sections of patients with MA-ARDS (10). Furthermore, lymphocytes and a small number of neutrophils are present (11). In murine MA-ARDS, especially CD8^+^ T cells are pathogenic (9). Recent studies also suggested neutrophils to play a detrimental role, whereas monocytes appeared rather protective by phagocytosing infected erythrocytes (12, 13). Currently, adequate treatment for MA-ARDS is not available (2, 9).

Glucocorticoids (GCs) are among the most effective therapy prescribed for various inflammatory diseases including asthma, allergy, and rheumatoid arthritis (14–16). GCs exert their anti-inflammatory and immunosuppressive effects mainly *via* the glucocorticoid receptor (GR) which belongs to the superfamily of ligand-inducible transcription factors (17). Activated GR can bind to specific DNA motifs (glucocorticoid response elements, GREs) and transactivate gene transcription. GR interacts with DNA as dimers or monomers. Endogenous GCs favor monomeric GR interactions with half-site motifs. GR monomers bound to half sites in liver and macrophages induce transcription and determine tissue-specific actions of GR. In contrast, exogenous GCs regulate gene expression *via* GR homodimers binding to classic palindromic motifs (18). Moreover, GCs can inhibit gene transcription *via* another mechanism called transrepression, which largely depends on protein–protein interactions (19–22). Transrepression represents the main mechanism by which GCs inhibit proinflammatory transcription factors such as NF-κB and AP-1 (23).

Since macrophages, monocytes, epithelial cells, and endothelial cells play different roles in the pathogenesis of ARDS, it is crucial to define the cell-specific mode of GC actions. In particular, recent reports suggest that macrophages are indispensable for initiation and termination and of lung inflammation. Moreover, these cells initiate the repair process (24). For example, Vettorazzi et al. showed that macrophages play an important role in the resolution of lung inflammation in animal models of acute lung injury. In macrophages, GCs synergize with proinflammatory stimuli to upregulate sphingosine kinase 1 (SphK1) and its enzymatic product, sphingosine 1-phosphate (S1P)—the major regulators of endothelial barrier integrity in the lungs (25). Furthermore, anti-inflammatory actions of GCs in the lungs depend on the circadian system (26). GCs also exert essential effects on endothelial cells by regulating multiple processes such as cytokine and chemokine expression and barrier permeability (27).

Although GCs remain an efficient therapy for inflammatory diseases, a subset of patients show poor or no response to GC therapy (15). GC resistance remains one of the major drawbacks of GC treatment and has been observed in patients suffering from diseases such as asthma, chronic obstructive pulmonary disease, and leukemia, and in various immune cells including peripheral blood mononuclear cells (PBMCs), B cells, and alveolar macrophages (14, 28). A variety of molecular mechanisms leads to GC resistance, e.g., increased GRβ expression, reduced GR translocation and impaired transactivation (29–31). Moreover, recent studies have confirmed that the endothelium represents an important target for GCs and several mechanisms of GC resistance in endothelial cells, including proteasomal degradation and epigenetic modifications of GR were reported (27, 32, 33).

We previously developed an *in vivo* model of MA-ARDS using C57BL/6 mice infected with *Plasmodium berghei* NK65 (*Pb*NK65) (9). These mice develop MA-ARDS with increased vascular permeability, protein-rich lung edema, and leukocyte infiltration. According to the observed histopathology, this mouse model exhibits important similarities to the human MA-ARDS and is suitable to investigate both pathogenesis and therapeutic strategies (2). High doses of GCs failed to inhibit expression of several proinflammatory cytokines in the lungs of these mice suggesting that malaria decreases GC sensitivity (9). GC resistance may also explain why clinical trials against cerebral malaria with dexamethasone were unsuccessful (34, 35). The mechanisms underlying GC resistance in this model of MA-ARDS remain unknown. In this study, we established an *in vitro* model of MA-ARDS with L2 microvascular lung endothelial cells and we aimed to delineate mechanisms of GC resistance in MA-ARDS.

## 2 Materials and Methods

### 2.1 Cell Cultures

The murine lung microvascular endothelial cell line (L2 MVEC) was cultured in RPMI medium (Gibco, Belgium) supplemented with 2 mM l-glutamine (Gibco), 0.1 mg/mL streptomycin (Sigma, Belgium), 200 U/mL penicillin (Kela, Belgium), and 10% FCS (Gibco). Cells were grown in 5% CO_2_ at 37°C.

### 2.2 Mice

All animal experiments were performed in accordance to the regulations as declared in Directive 2010/63/EU from the European Union and the Belgian Royal Decree of May 29, 2013, and were approved by the Animal Ethics Committee from the KU Leuven (project number P163-2014, License LA1210186, Belgium). All efforts were made to minimize suffering of animals. Unless otherwise indicated, male Balb/c mice were obtained from Janvier (7–8 weeks old, Le Genest-Saint-Isle, France). Mice were injected intraperitoneally with 10^6^ *Pb*NK65-infected red blood cells (a kind gift of the late Prof. D. Walliker, University of Edinburgh). Mice were kept in a conventional animal house and drinking water was supplemented with 4-amino benzoic acid (0.375 mg/mL, PABA, Sigma-Aldrich, Bornem, Belgium). Parasitemia was determined by microscopic analysis of tail blood smears after Giemsa staining (1/10 dilution, VWR, Heverlee, Belgium). Mice were sacrificed 8 days after infection (when parasitemia was approximately 4%) by euthanasia with Dolethal (Vtoquinol, Aartselaar, Belgium; 200 mg/mL, intraperitoneal injection of 50 µL) and cardiac punctures were performed.

### 2.3 *Ex Vivo* Cultivation of *Pb*NK65 and Extract Production

After cardiac punctures, blood was filtered with Plasmodipur filters (Europroxima) to remove leukocytes and washed with RPMI medium (Gibco) supplemented with 25 mM HEPES, 0.425 g NaHCO_3_, 2 mM l-glutamine (Gibco), 5 mM glucose (Sigma), and 20% FCS. The pellet was resuspended and seeded into culture flasks. Cells were gassed with a mixture of 92.5% N_2_, 5.5% CO_2_, 2% O_2_ and cultured overnight at 37°C. The next day the cultures were centrifuged, resuspended in RPMI medium and loaded on MACS columns (Miltenyi Biotec, NL) to purify the schizonts. After elution the schizonts were diluted to the concentration of 10^8^/mL in RPMI medium, aliquoted, and frozen at −20°C. Next they were thawed and frozen at −20°C again to produce the extract.

### 2.4 Stimulation of L2 MVEC Cells with IFN-γ, *Pb*NK65 Extract, and MAPK Inhibitors

L2 MVECs were seeded in 6-, 24-, 48-, or 96-well plates at the concentration of 5 × 10^4^ cells/mL. After expanding the cells for 24 h, they were washed with medium and stimulated for 24 h with combinations of murine IFN-γ (20 ng/mL, PeproTech, USA), parasite extract (10^7^ infected red blood cells—iRBCs/mL), murine red blood cells extract (10^7^ RBCs/mL), and dexamethasone (100 nM dissolved in DMSO, Sigma). JNK, p38, and ERK inhibitors (SP600125, SB203580, and FR180204, dissolved in DMSO, R&D, UK) were used at 20, 5, and 10 µM, respectively. After stimulation, plates were centrifuged (5 min, 1,200 rpm, RT) and supernatants were collected for ELISA and stored at −20°C. Cytokines were analyzed by ELISA (R&D). Cells were washed with PBS, lysed with RLT buffer with β-mercaptoethanol from RNeasy Mini kit (Qiagen, Belgium), and stored at −80°C for RNA extraction.

### 2.5 Quantitative RT-PCR and RNA-Seq

RNA was extracted with RNeasy Mini Kit (Qiagen) according to the manufacturer’s protocol. RNA concentration and purity were evaluated with Nanodrop 1000 (Thermo Scientific, Belgium). RNA (0.25 µg) was converted to cDNA using high-capacity cDNA reverse transcription kit (Applied Biosystems). Quantitative RT-PCR was performed with 6.25 or 0.125 ng cDNA using predesigned primers (IDT) and TaqMan Universal PCR Master Mix (Applied Biosystems), respectively. RNA-Seq expression profiling was performed by the Genomics Core UZ Leuven. Per independent experiment RNA from three technical replicates per experimental condition was pooled and 3 µg RNA/experimental condition were sequenced from a total of three independent experiments. Illumina TruSeq stranded mRNA kit was used and the single-end sequencing was performed. 33 M 50 bp reads per sample were sequenced. Reads were aligned to mm10 murine genome using TopHat. A heatmap with highly variable genes across the samples was plotted using pheatmap package (pheatmap). The rlog transformed counts of each gene were centered across the samples. Ribosomal RNA genes and predicted genes that were increased in one sample (GC027190) were excluded from the rlog data used for heatmap since this increase was a result of an imperfect poly-A selection during the library preparation of this sample. Differential expression analysis was performed with DESeq2 (36). Differences in gene expression with a FDR adjusted *p* value below 0.1 were considered significant. Gene ontology analysis of differentially expressed genes was performed with clusterProfiler package (37). Motif analysis of the proximal promoter region (400 bp upstream of the transcription start site till 100 bp downstream) was performed using Homer software (Homer motif analysis). RNA-seq data were submitted to ArrayExpress (accession number E-MTAB-5921 (ArrayExpress URL)).

### 2.6 Western Blot

For Western blot analysis, protein extracts from L2 MVECs stimulated with IFN-γ, *Pb*NK65 extract, IFN-γ and *Pb*NK65 extract in the presence or absence of dexamethasone were separated on SDS PAGE gels and blotted onto a PVDF membrane. Blocking was performed with BSA (Carl Roth Gmbh, Belgium) or non-fat dry milk (Bio Rad, USA). The following specific primary Abs were used: JNK, pJNK, p38, p-p38 (1:2,000, Cell Signaling Technology, The Netherlands), p-GR S211 (1:1,000, Cell Signaling Technology), p-GR S226 (1:1,000, Abcam), GR H300 (1:1,000, Santa Cruz, Germany), p-STAT1 Tyr701 (1:1,500, Cell Signaling Technology), and STAT1 (1:5,000, Cell Signaling Technology). Fusion solo S system (Vilber, France) was used to take chemiluminescence Western blot images. Quantification of Western blot images was performed by densitometry (ImageJ software was used).

### 2.7 GR Nuclear Translocation

Cells were seeded on coverslips and incubated in phenol-red-free and serum-free medium for 4 h. Cell fixation, methanol permeabilization, and staining were performed according to Cell Signaling guidelines. GR was visualized with the GR polyclonal (H300) antibody (Santa Cruz, Germany), used at 1:200, followed by probing with Alexa Fluor 488 (Invitrogen, Belgium). Nuclei were visualized using 4′,6-diamidino-2-phenylindole (DAPI) staining. A motorized inverted IX81 FluoView FV1000 laser scanning confocal microscope (Olympus) was used to record high-resolution images. Assessment of intracellular localization of protein signal was done double blind.

### 2.8 Statistical Analysis

Statistical analysis was performed with R (The R Project for Statistical Computing). Mann–Whitney or ANOVA was used to analyze ELISA and qPCR data. Statistical significance was set at a *p* value below 0.05.

## 3 Results

### 3.1 Lung Endothelial Cells Remain GC Sensitive upon Stimulation with IFN-γ

**Figure 1:**
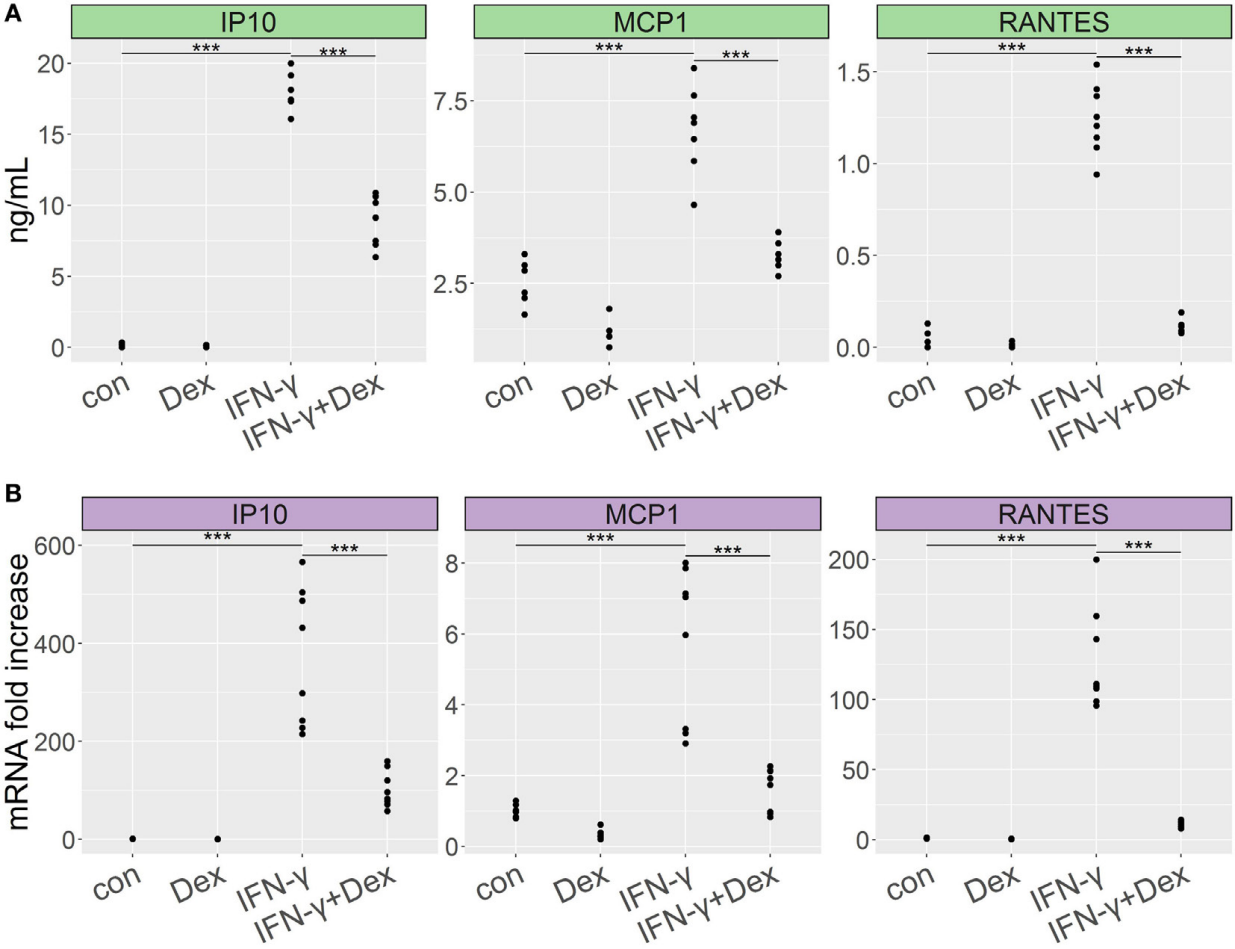
Dexamethasone inhibits proinflammatory cytokine secretion in lung endothelium stimulated with IFN-γ. L2 MVECs were treated with vehicle (con) or IFN-γ (20 ng/mL) in the presence or absence of dexamethasone (Dex, 100 nM) for 24 h. CCL2 (MCP-1), CXCL10 (IP-10), and CCL5 (RANTES) expression levels were analyzed by ELISA **(A)** and real-time qPCR **(B)**. Statistical significance was evaluated using ANOVA (***p < 0.001). Data show combined results from three independent experiments.

IFN-γ plays a crucial role in the induction of chemokines in mouse models of complicated malaria (38, 39). To evaluate whether IFN-γ might cause GC resistance in lung endothelial cells, we stimulated L2 MVECs with IFN-γ in the presence or absence of dexamethasone for 24 h. IFN-γ induced CC chemokine ligand 2 (CCL2, also known as monocyte chemoattractant protein 1 or MCP-1), CXC chemokine ligand 10 (CXCL10, also known as IFN-γ-induced protein 10 or IP-10), and CCL5 (also known as regulated on activation normal T cell expressed and secreted or RANTES) on both protein and RNA levels (Figure 1). CXCL10 (IP-10) showed the strongest induction. Dexamethasone inhibited IFN-γ-induced chemokine secretion, suppressing CCL5 (RANTES) by 90% and both CXCL10 (IP-10) and CCL2 (MCP-1) approximately by 50% (Figure 1A). Real-time qPCR experiments revealed 90% inhibition of CCL5 (RANTES) and 71% inhibition of both CXCL10 (IP-10) and CCL2 (MCP-1). Moreover, treatment with dexamethasone (with or without IFN-γ) induced DUSP-1 (MKP1), GILZ (TSC22d3), and FKBP51 (Figure S1 in Supplementary Material), indicating that IFN-γ did not alter GC-mediated transactivation of these genes. These results indicate that IFN-γ-stimulated lung endothelial cells remain GC sensitive.

### 3.2 GCs Differentially Affect the Transcriptional Response to IFN-γ in Lung Endothelial Cells

**Figure 2:**
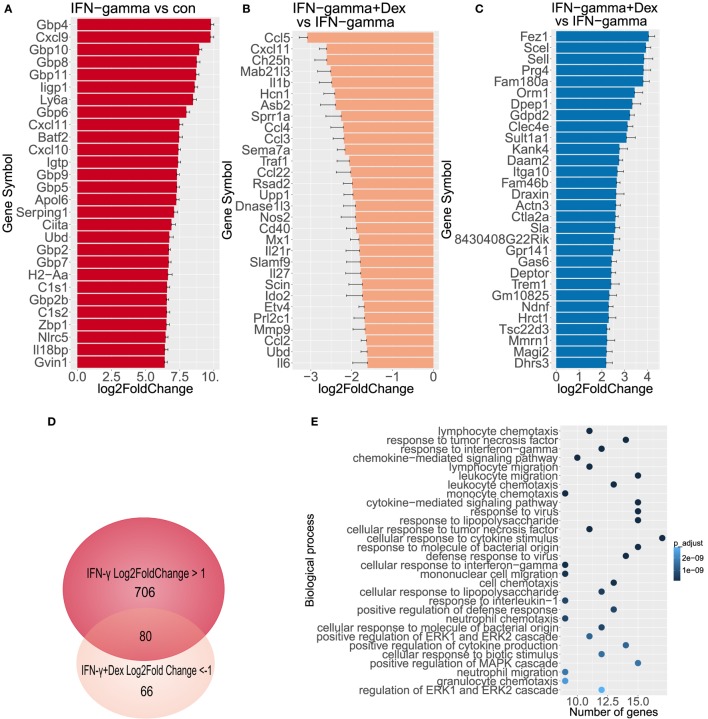
GCs diminish transcriptional activity induced by IFN-γ. Genome wide expression was measured (n = 3), log2FoldChange has been depicted including standard error estimate (lfcSE, error bars). **(A)** Top 30 genes activated by IFN-γ in endothelial cells. **(B)** Top 30 genes downregulated by dexamethasone in endothelial cells stimulated with IFN-γ. **(C)** Top 30 genes induced by dexamethasone in IFN-γ-stimulated endothelial cells. **(D)** Venn diagram depicts overlap between genes induced by IFN-γ and repressed by dexamethasone. **(E)** Biological process GO analysis (top 30 terms) of genes induced at least twofold by IFN-γ and repressed at least 50% by dexamethasone.

To further characterize the transcriptional targets indicating GC sensitivity in lung endothelial cells, we analyzed by RNA-Seq the transcriptome of L2 MVECs stimulated with IFN-γ in the presence or absence of dexamethasone. IFN-γ induced expression of various guanylate binding proteins (GBPs, Figure 2A), which trigger antimicrobial effector mechanisms *via* inhibition of replication of intracellular pathogens (40). Similarly, we found several interferon-induced proteins with tetratricopeptide repeats (IFITs, Supplementary File 1). These proteins inhibit viral replication (41). IFN-γ also increased expression of H-2 class II histocompatibility genes and adhesion molecules such as vascular cell adhesion protein 1 (VCAM-1) and intercellular adhesion molecule 1 (ICAM-1, Supplementary Files 1 and 2). Moreover, IFN-γ potently induced transcription of various proinflammatory CXC and CCL chemokines including CXCL9 (also known as monokine induced by gamma interferon or MIG), CXCL11 (also known as Interferon-inducible T cell alpha chemoattractant or I-TAC), CXCL10 (IP-10), CCL5 (RANTES), and CCL2 (MCP-1, Figure 2A; Table 1; Supplementary Files 1). These data on chemokine induction further confirm the ELISA and qRT-PCR experiments shown in Figure 1.

**Table 1:** Differentially expressed chemokines and cytokines in lung endothelial cells stimulated with IFN-γ in the presence or absence of dexamethasone.

Gene symbol	IFN-γ vs. con (Log2FC)	IFN-γ + Dex vs. IFN-γ (Log2FC)
CXCL16	2.4	−1.2
CXCL14	−1.7	0.75
CXCL12	−0.8	1.2
CXCL11	7.5	−2.6
CXCL10	7.4	−1.6
CXCL9	9.7	−1.6
CXCL5	NS	−1.4
CXCL1	NS	−1.2
CCL27a	0.8	NS
CCL22	3.9	−2
CCL20	1.4	−1.5
CCL17	NS	−1
CCL12	3.8	−1.4
CCL11	1.9	NS
CCL9	−1.3	0.9
CCL8	3.3	−0.9
CCL7	1.6	−1.4
CCL6	1.2	NS
CCL5	6	−3.1
CCL4	2.4	−2.2
CCL3	1.1	−2.2
CCL2	2.2	−1.6
IL-34	NS	−1.1
IL-33	−1.5	NS
IL-27	3.4	−1.8
IL-18	0.6	0.6
IL-15	2.4	−0.5
IL-10	1.4	1.4
IL-7	1.2	NS
IL-6	0.8	−1.6
IL-1*α*	2.5	−1.6
IL-1β	4.2	−2.5
TNF	2.2	−1.6
TNFSF18	1	NS
TNFSF15	2.1	−1.6
TNFSF13b	2.8	NS
TNFSF11	−0.8	NS
TNFSF8	−1.3	NS

*FC, fold change; NS, non-significant*.

Dexamethasone significantly inhibited large clusters of IFN-γ-induced genes, which included several CXC and CCL chemokines (Table 1; Supplementary Files 1 and 3). In particular, dexamethasone markedly diminished transcription of CCL5 (RANTES, more than 85% reduction), CXCL11 (I-TAC, more than 80% reduction), CCL4, and CCL3 (more than 75% reduction, Figure 2B). Also, IFN-γ-induced interleukin 1β (IL-1β), IL-27, and IFITs were downregulated by dexamethasone. In contrast, the expression of GBPs, signal transducer and activator of transcription (STAT) family members, and the cytokine IL-15 was resistant to GC-mediated transcriptional inhibition (Supplementary File 1).

In addition, we found a large cluster of genes upregulated by GCs (Supplementary File 1). Dexamethasone induced several anti-inflammatory genes in IFN-γ-stimulated endothelial cells including orosomucoid-1 (Orm1, Fold Change 11.3), DEP domain-containing mTOR-interacting protein (DEPTOR, Fold Change 5.3), and TSC22 domain family, member 3 (TSC22d3 also known as GILZ, Fold Change 4.6, Figure 2C)—a known GR target gene. These genes were also upregulated upon treatment with dexamethasone alone (Supplementary File 4). Interestingly, another set of genes was repressed by IFN-γ alone, but the expression was restored when dexamethasone was added. These genes include CXCL12, Angptl7, TGF-β2, TLR7, and TNFRSf21 (Supplementary File 1). Altogether, these data indicate that lung endothelial cells are GC sensitive, when stimulated with IFN-γ. Furthermore, these cells provide a novel model to study GR-dependent responses.

As for the affected pathways, gene ontology (GO) analysis of genes induced by IFN-γ at least 2-fold and inhibited by dexamethasone at least by 50% (Figure 2D; Supplementary File 5) revealed a high prevalence of biological process GO terms related to regulation of immune response and proinflammatory signaling pathways such as MAPK (Figure 2E). Several binding partners have been involved in GR-mediated repression of proinflammatory genes including AP-1, NF-κB, and IRF3 (42–44). Interestingly, a computational analysis of the 400-bp region upstream of genes induced by IFN-γ at least 2-fold and inhibited by dexamethasone at least by 50% showed a significant enrichment of the binding sites for IRF and NF-κB families but not for GR (Table S1 in Supplementary Material). These results remain in line with previous findings since in LPS-stimulated macrophages, less than 6% of GR binding sites occurred at proximal promoter regions (44). Also, GR can tether to DNA-bound TFs such as NF-κB without requiring a GRE motif.

### 3.3 *Pb*NK65 Extract in Combination with IFN-γ Induces GC Resistance in Lung Endothelial Cells

**Figure 3:**
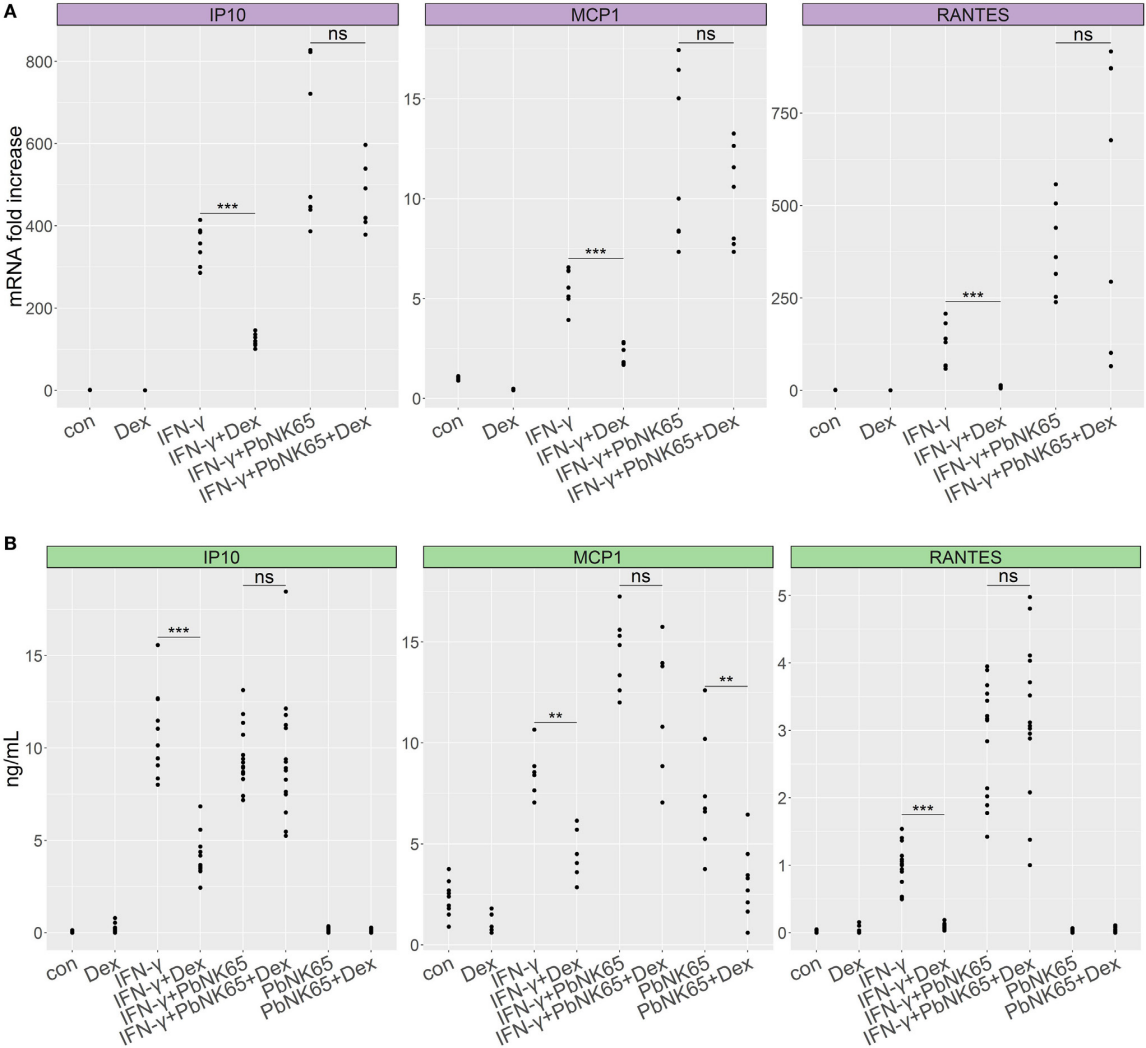
*Pb*NK65 extract in combination with IFN-γ induces GC resistance in lung endothelial cells. L2 MVECs were treated with vehicle (con), IFN-γ (20 ng/mL), *Pb*NK65 extract or IFN-γ and *Pb*NK65 extract (PbNK65, 10^7^ infected RBCs/mL) in the presence or absence of dexamethasone (Dex, 100 nM) for 24 h. CCL2 (MCP-1), CXCL10 (IP-10), and CCL5 (RANTES) levels were analyzed by real-time qPCR **(A)** and ELISA **(B)**. Statistical significance was evaluated using Mann–Whitney test (**p < 0.01, ***p < 0.001). Data represent combined results from at least two independent experiments.

Although GC resistance has been observed in our murine model of MA-ARDS (9), the above results indicate that IFN-γ-stimulated lung endothelial cells remain GC sensitive. As sequestering parasites release a variety of products which can further activate endothelial cells (6, 7), we investigated whether addition of parasite extract might alter the GC sensitivity of L2 MVECs. *Pb*NK65 extract in combination with IFN-γ increased the mRNA levels of CCL2 (MCP-1) and CCL5 (RANTES), respectively, 2-fold and 3-fold in comparison to IFN-γ alone (Figure 3A). *Pb*NK65 alone increased only CCL2 (MCP-1) protein levels and interestingly, this induction was sensitive to dexamethasone (Figure 3B). Importantly, dexamethasone failed to inhibit mRNA and protein induction of CCL2 (MCP-1), CCL5 (RANTES), and CXCL10 (IP-10), when cells were challenged with the combination of *Pb*NK65 extract and IFN-γ (Figures 3A,B). These results indicate that *Pb*NK65 extract impairs GC-mediated transrepression of these inflammatory chemokines observed after stimulation with IFN-γ alone. In contrast, *Pb*NK65 extract did not decrease GC-induced transactivation of DUSP-1 (MKP1), GILZ (TSC22d3), and FKBP51 (Figure S1 in Supplementary Material).

To evaluate the time course of the development of GC resistance, we stimulated lung endothelial cells with *Pb*NK65 extract and IFN-γ for 6, 24, and 48 h in the presence or absence of dexamethasone and analyzed CCL2 (MCP-1) and CXCL10 (IP-10) secretion. After 6 h, chemokine levels remained low both in the resistant condition with *Pb*NK65 extract and IFN-γ and in the sensitive one with IFN-γ alone (Figure S2 in Supplementary Material). GC resistance for CCL2 (MCP-1) occurred following 24-h stimulation with *Pb*NK65 extract and IFN-γ and was still present after 48 h (Figure S2A in Supplementary Material). *Pb*NK65 extract and IFN-γ induced GC resistance for CXCL10 (IP-10) already after 6 h (Figure S2B in Supplementary Material). Notwithstanding these gene-specific differences in kinetics, all studied genes underwent GC resistance upon combining IFN-γ with *Pb*NK56 extract. In contrast, upon stimulation with IFN-γ alone endothelial cells remained fully GC sensitive even after 48 h (Figures S2C,D in Supplementary Material).

**Figure 4:**
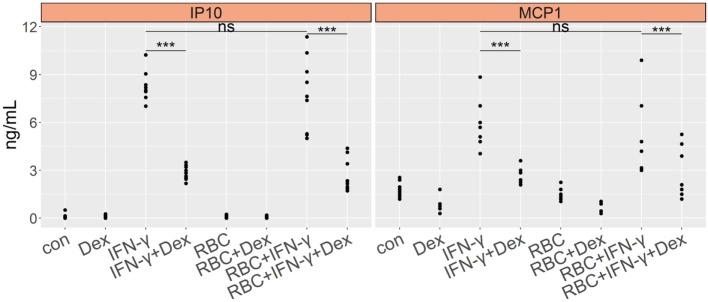
Murine red blood cell extract (RBC) exhibits no effect on cytokine induction and GC sensitivity of lung endothelial cells. L2 MVECs were stimulated with vehicle (con), IFN-γ (20 ng/mL), RBC (10^7^ RBCs/mL), RBC and IFN-γ in the presence or absence of dexamethasone (Dex, 100 nM) for 24 h. CCL2 (MCP-1) and CXCL10 (IP-10) production was analyzed by ELISA. Statistical significance was evaluated using ANOVA (***p < 0.001). Data represent combined results from 2 independent experiments.

As a control for the *Pb*NK65 extract, we stimulated lung endothelial cells with extract from non-infected red blood cells (RBC) and evaluated GC sensitivity. RBC extract did not induce CCL2 (MCP-1) or CXCL10 (IP-10) in endothelial cells (Figure 4). When combined with IFN-γ, RBC extract failed to enhance secretion of proinflammatory chemokines. Furthermore, dexamethasone inhibited CCL2 (MCP-1) and CXCL10 (IP-10) production induced by IFN-γ in the presence of RBC extract, showing that RBC extract is not able to induce GC resistance. These results confirm that parasite components but not RBC components mediate GC resistance.

### 3.4 *Pb*NK65/IFN-γ Cotreatment Preserves Homologous GR Downregulation and GR Nuclear Translocation

**Figure 5:**
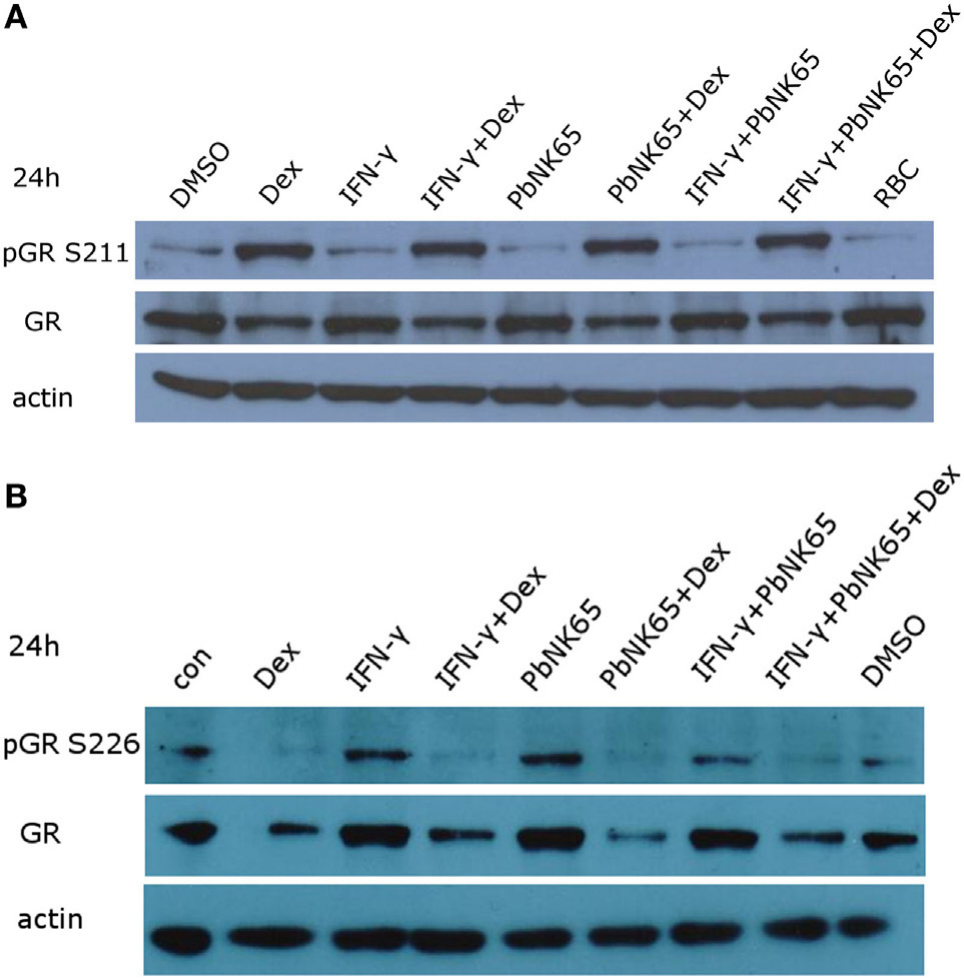
IFN-γ and *Pb*NK65 extract do not interfere with either GR expression or GR phosphorylation. **(A,B)** Western blot analysis of lysates of L2 MVECs stimulated for 24 h with solvent (DMSO), red blood cells extract (RBC, 10^7^ RBCs/mL), IFN-γ (20 ng/mL), *Pb*NK65 extract (10^7^ infected RBCs/mL), IFN-γ and *Pb*NK65 extract in the presence or absence of dexamethasone (Dex, 100 nM).

GR undergoes homologous downregulation when incubated with its ligand for longer periods of time. Perturbations of this process may be lead to GC resistance (45, 46). To evaluate the levels of GR, we stimulated L2 MVECs for 6 or 24 h with vehicle (DMSO), red blood cell extract (RBC), IFN-γ, *Pb*NK65 extract, IFN-γ and *Pb*NK65 extract in the presence or absence of dexamethasone. The levels of GR remained unchanged in the resistant condition with IFN-γ and *Pb*NK65 extract when compared to the sensitive condition with IFN-γ alone. Additionally, the capacity and extent of GC-induced GR homologous downregulation remained unaffected in either the sensitive or resistant condition. We also investigated the serine-211 (S211) phosphorylation of GR, since this modification is associated with transcriptionally active GR and provides a means for cross-talk with other signaling pathways (47). As expected, dexamethasone alone induced S211 phosphorylation. Moreover, phosphorylation of GR at S211 remained present when cells were challenged with IFN-γ or IFN-γ with *Pb*NK65 extract in the presence of dexamethasone (Figure 5A; Figure S3 in Supplementary Material). We also evaluated the GR S226 phosphorylation, which inhibits GR function (48, 49). Dexamethasone inhibited GR S226 phosphorylation upon stimulation with IFN-γ or IFN-γ with *Pb*NK65 extract (Figure 5B). These data indicate that GC resistance following IFN-γ/*Pb*NK65 cotreatment is most likely not caused by defective GR phosphorylation or GR homologous downregulation mechanisms.

**Figure 6:**
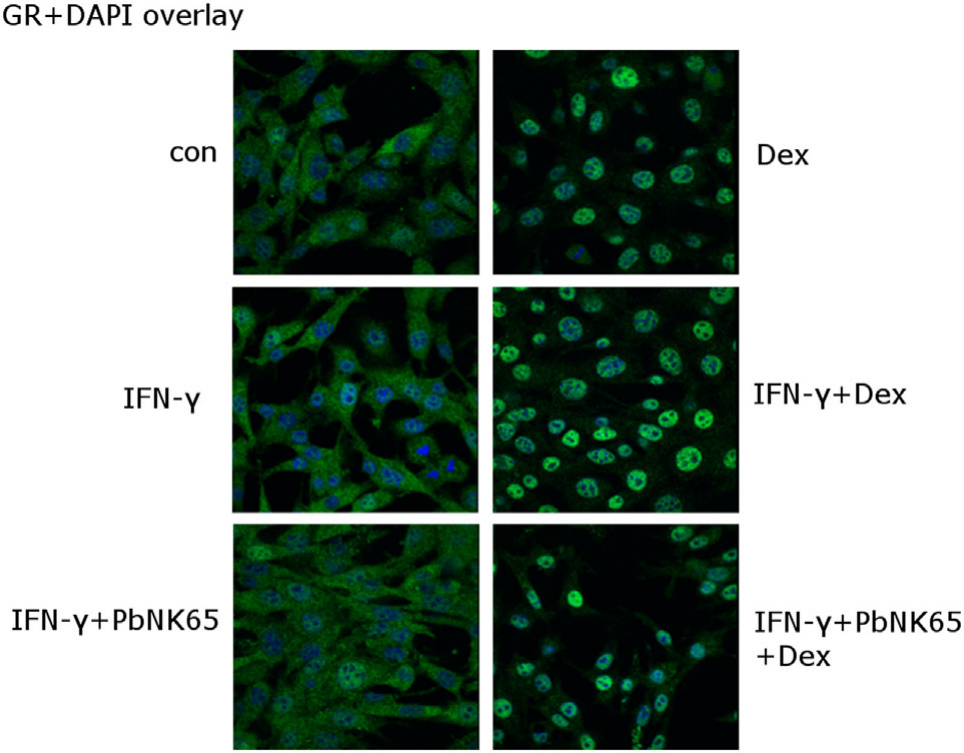
IFN-γ and *Pb*NK65 extract preserve GR nuclear translocation in lung endothelial cells. L2 MVECs were stimulated with solvent (con), IFN-γ (20 ng/mL) or IFN-γ and *Pb*NK65 extract (10^7^ infected RBCs/mL) for 24 h and treated for 1 h with dexamethasone (100 nM). Endogenous GR was visualized (green) through indirect immunofluorescence using anti-GR Ab. DAPI staining (blue) indicates the nuclei of the cells.

Since impaired GR nuclear translocation leads to GC resistance (31, 50), we assessed the ability of dexamethasone to induce GR translocation in lung endothelial cells. L2 MVECs were challenged for 24 h with *Pb*NK65 extract and IFN-γ and dexamethasone was added during the last hour of stimulation (Figure 6). Immunofluorescence microscopy revealed that in unstimulated cells GR localized mainly in the cytoplasm and translocated to the nucleus upon dexamethasone exposure. GR also translocated to the nucleus upon treatment with IFN-γ and dexamethasone. Furthermore, the translocation occurred in the GC resistant condition with *Pb*NK65 extract and IFN-γ (Figure 6). We also obtained similar data for a shorter time point of 2 or with 24 h cotreatment with IFN-γ, *Pb*NK65 extract and dexamethasone (Figures S4A,B in Supplementary Material). These data indicate that GC resistance is not caused by any defect in GR translocation.

### 3.5 Dexamethasone Fails to Inhibit STAT1 Activation upon Challenge with IFN-γ or *Pb*NK65 Extract and IFN-γ

**Figure 7:**
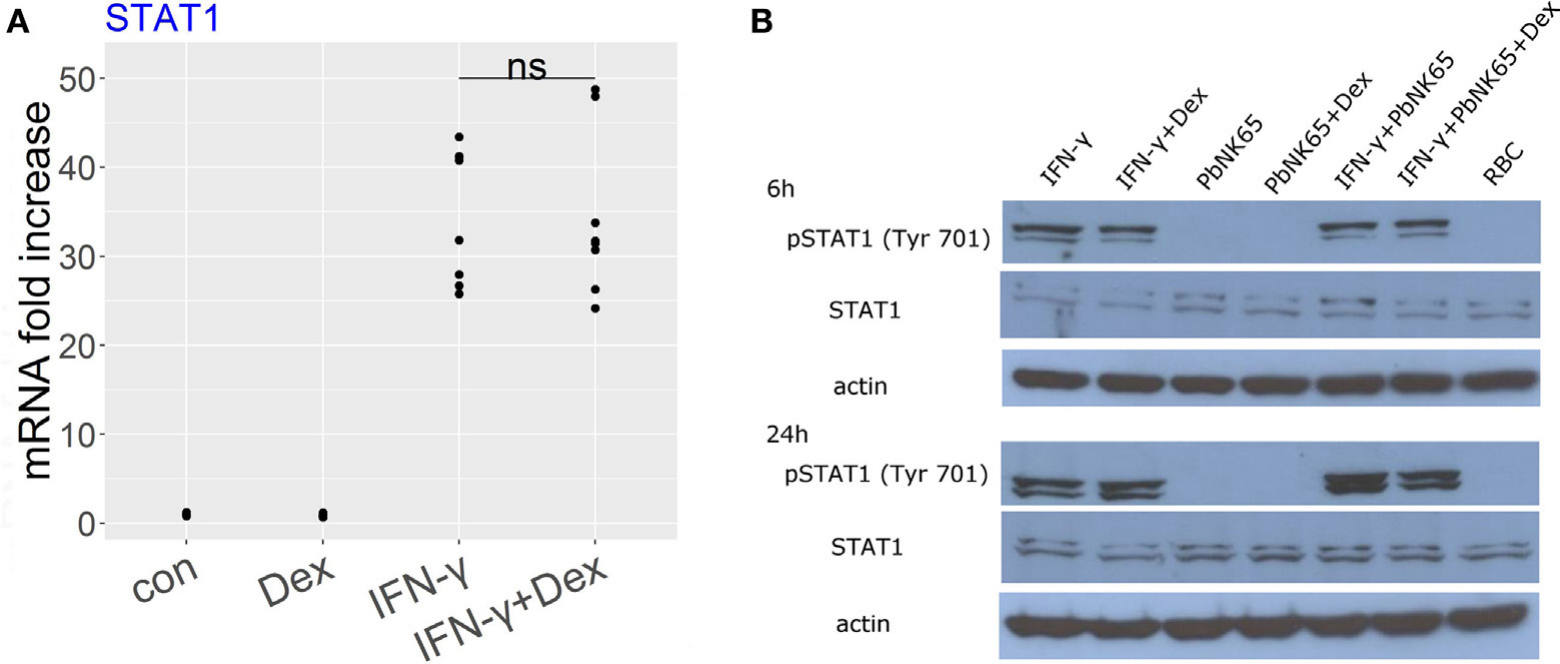
Dexamethasone fails to inhibit STAT1 expression or STAT1 phosphorylation induced by IFN-γ or IFN-γ and *Pb*NK65 extract. **(A)** L2 MVECs were stimulated with solvent (con) and IFN-γ (20 ng/mL) in the presence or absence of dexamethasone (Dex, 100 nM) for 24 h. STAT1 expression was analyzed by qRT-PCR. Statistical significance was evaluated using Mann-Whitney test. Data show combined results from three independent experiments. **(B)** Western blot analysis of lysates of L2 MVECs stimulated for 6 or 24 h with IFN-γ (20 ng/mL), *Pb*NK65 extract (10^7^ infected RBCs/mL), IFN-γ and *Pb*NK65 extract in the presence or absence of dexamethasone (Dex, 100 nM) and red blood cell extract (RBC, 10^7^ RBCs/mL) was performed using anti-STAT1 and anti-pSTAT1 Ab.

Since IFN-γ signals *via* STAT1 to induce gene transcription, we evaluated the effect of dexamethasone on STAT1 activation in the GC sensitive condition with IFN-γ and in the resistant one with IFN-γ and *Pb*NK65 extract. RNA-Seq data showed that dexamethasone failed to affect STAT1 expression induced by IFN-γ and this result was validated by qRT-PCR (Figure 7A). Since STAT1 phosphorylation at Tyr 701 controls STAT1 signaling, we subsequently addressed the impact of dexamethasone on phosphorylated STAT1. We showed that STAT1 phosphorylation induced by IFN-γ remained unaffected by dexamethasone both in the GC sensitive (IFN-γ) and the GC resistant condition (IFN-γ and *Pb*NK65 extract, Figure 7B).

### 3.6 IFN-γ and *Pb*NK65 Extract Impair GC-Mediated Inhibition of MAPK Signaling

**Figure 8:**
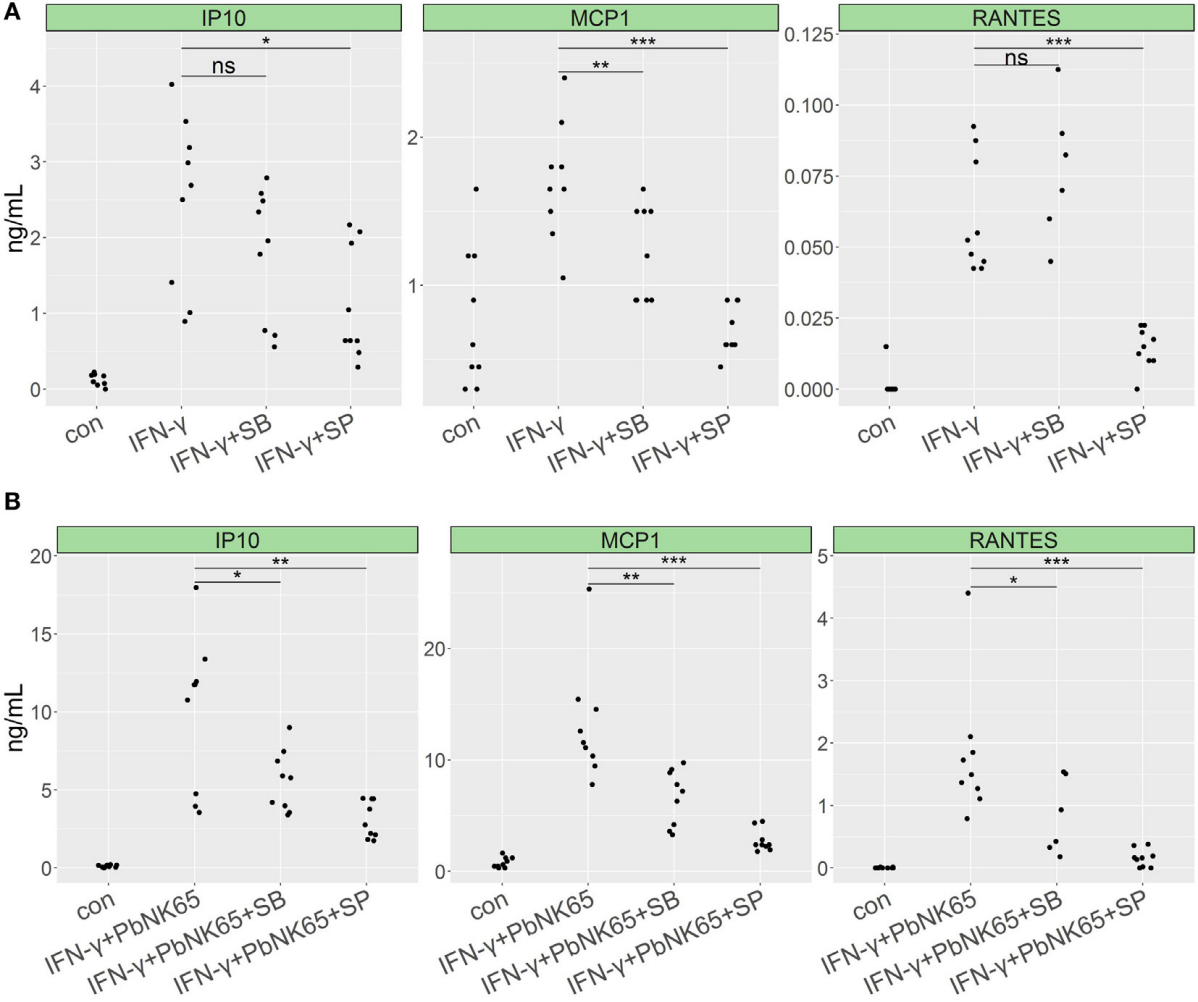
p38 and JNK inhibitors impair proinflammatory cytokine expression in lung endothelial cells. L2 MVECs were stimulated with IFN-γ (20 ng/mL) **(A)** or IFN-γ (20 ng/mL) and *Pb*NK65 extract (*Pb*NK65, 10^7^ infected RBCs/mL) **(B)** in the presence of JNK inhibitor SP600125 (SP, 20 µM) or p38 inhibitor SB203580 (SB, 5 µM) for 24 h. Protein levels of CXCL10 (IP-10), CCL2 (MCP-1), and CCL5 (RANTES) were determined by ELISA. Statistical significance was evaluated using ANOVA (*p < 0.05, **p < 0.01, ***p < 0.001). Data represent combined results from three independent experiments.

MAPK family members play an important role in the generation and fine-tuning of inflammatory responses and are known to be activated by IFN-γ receptor signaling (51, 52). To address the role of specific members of the MAPK family in the induction of proinflammatory chemokines in our model, we used the JNK-, p38-, and ERK-specific inhibitors: SP600125, SB203580, and FR180204, respectively. Treatment with JNK and p38 inhibitors significantly reduced proinflammatory response induced by IFN-γ in lung endothelial cells. Inhibition of p38 reduced CCL2 (MCP-1) levels by 28%, whereas inhibition of JNK blocked CXCL10 (IP-10), CCL2 (MCP-1), and CCL5 (RANTES) by 55, 59, and 83%, respectively (Figure 8A). Accordingly, JNK and p38 inhibition blocked chemokine induction in the GC resistant condition upon combining IFN-γ with *Pb*NK65 extract. SB203580 inhibited CXCL10 (IP-10), CCL2 (MCP-1), and CCL5 (RANTES) levels by 44, 49, and 54%, whereas SP600125 reduced CXCL10 (IP-10), CCL2 (MCP-1), and CCL5 (RANTES) by 69, 79, and 94%, respectively (Figure 8B). Inhibition of ERK failed to reduce CXCL10 (IP-10) CCL2 (MCP-1) or CCL5 (RANTES) levels upon challenge with IFN-γ and *Pb*NK65 extract (Figure S6 in Supplementary Material).

**Figure 9:**
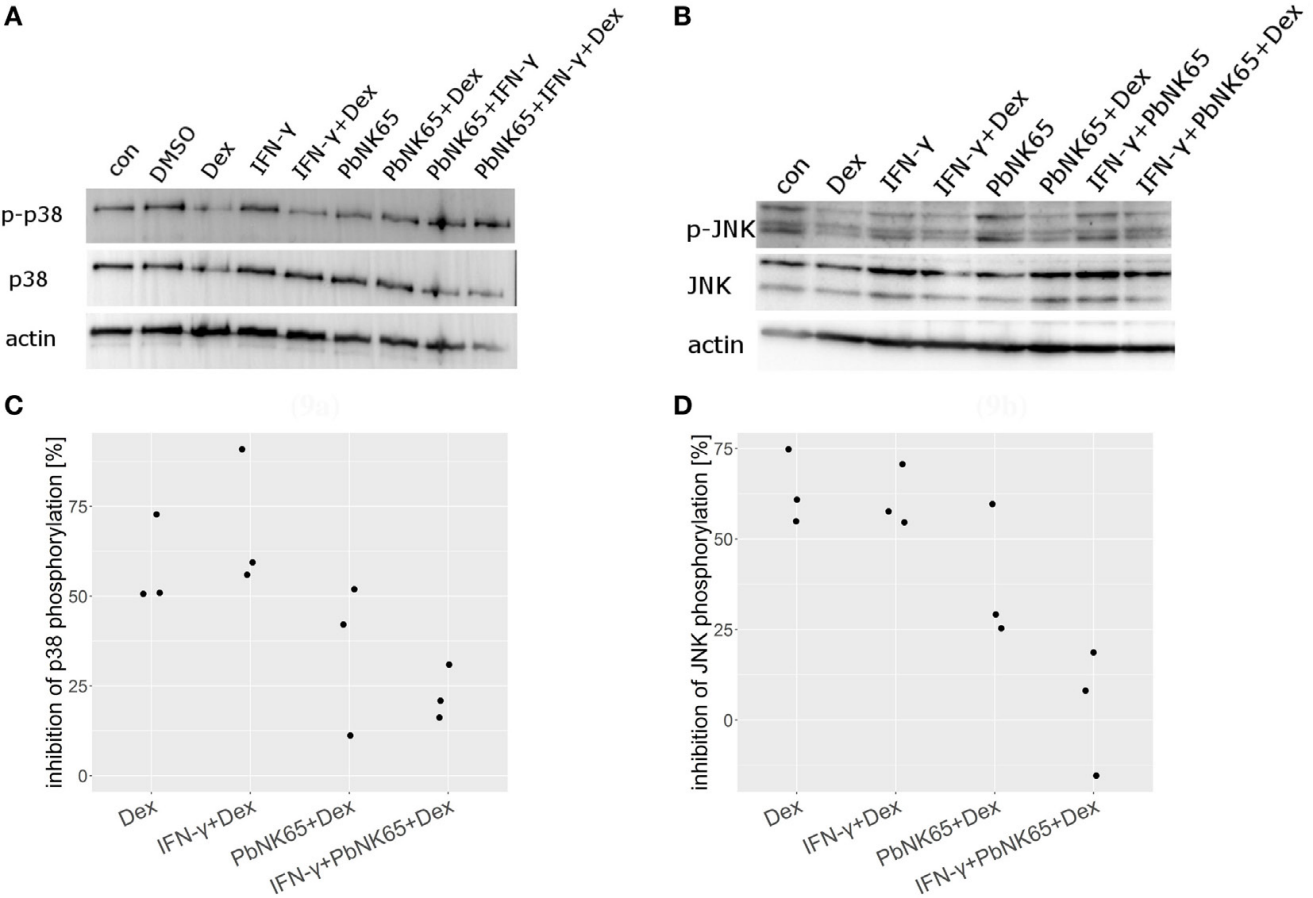
Dexamethasone fails to suppress JNK and p38 activation after challenge with IFN-γ and *Pb*NK65 extract. Western blot analysis of L2 MVEC lysates stimulated for 24 h with IFN-γ (20 ng/mL), IFN-γ and *Pb*NK65 (10^7^ infected RBCs/mL) extract in the presence or absence of dexamethasone (Dex, 100 nM) was performed using anti-p-p38, anti-p38 **(A)**, anti-pJNK, and anti-JNK **(B)** Ab. Data are representative of three independent experiments. **(C,D)** Data from three independent experiments were normalized against actin and percentage inhibition by dexamethasone was calculated.

Since previous studies indicated that GCs inhibit MAPK family members phosphorylation (53, 54), we investigated the effects of dexamethasone on p38 and JNK phosphorylation in endothelial cells stimulated with IFN-γ with and without *Pb*NK65 extract in the presence or absence of dexamethasone. We found that dexamethasone blocked p38 and JNK phosphorylation upon challenge with IFN-γ (Figures 9A,B). Quantification of the Western blot data revealed that, upon challenge with IFN-γ, dexamethasone reduced p38 and JNK phosphorylation on average by 69 and 61%. In unstimulated cells, dexamethasone also blocked p38 and JNK phosphorylation by 58 and 63%, respectively (Figures 9C,D). In contrast, when IFN-γ was combined with *Pb*NK65 extract dexamethasone failed to inhibit p38 and JNK phosphorylation by more than 14 and 4% (Figures 9A–D). These data suggest that dexamethasone inhibits IFN-γ-mediated induction of CXCL10 (IP-10), CCL2 (MCP-1), and CCL5 (RANTES) at least in part by blocking the activation of p38 and JNK. Furthermore, this inhibitory mechanism is impaired in the GC resistant condition.

## 4 Discussion

**Figure 10:**
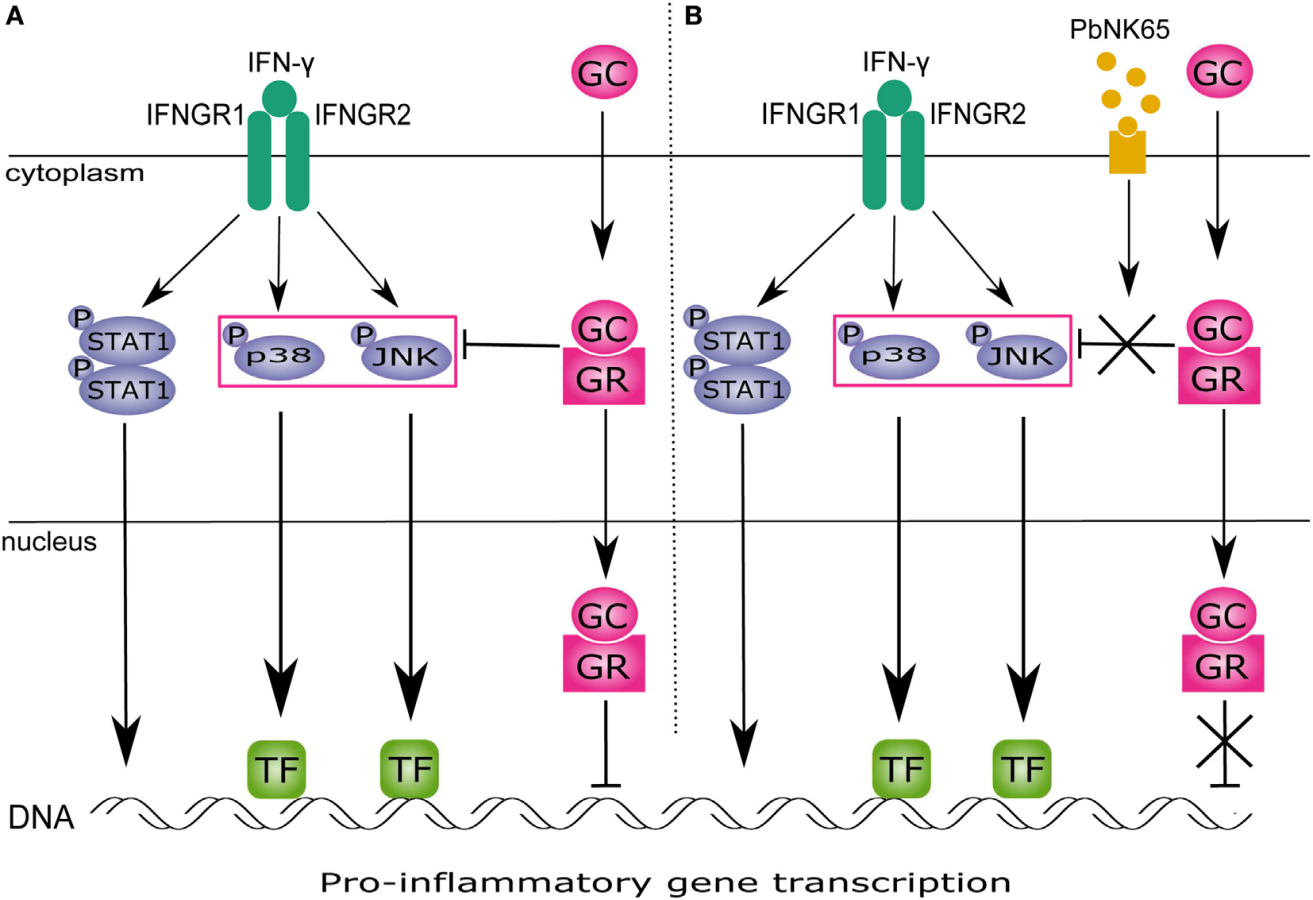
*Pb*NK65 extract in combination with IFN-γ impairs inhibitory effects of glucocorticoids (GC) on MAPK in lung endothelium. **(A)** IFN-γ signals through IFNGR and activates STAT1 and MAPK signaling. STAT1 homodimer translocates to the nucleus and activates gene transcription. MAPK induce gene expression *via* specific transcription factors (TFs). GC upon entering the cell bind to glucocorticoid receptor (GR) and inhibit activation of JNK and p38 induced by IFN-γ. This reduces proinflammatory genes expression. **(B)** When cells are stimulated with IFN-γ and *Pb*NK65 extract, GC fail to block p38 and JNK activation. Therefore, transcription of proinflammatory genes remains unaffected.

In this study, we show that *Pb*NK65 extract in combination with IFN-γ induces GC resistance in lung endothelial cells. GCs block JNK and p38 activation and proinflammatory chemokine release upon challenge with IFN-γ. However, stimulation with *Pb*NK65 extract in combination with IFN-γ impairs the ability of GCs to block JNK and p38 signaling (Figure 10).

IFN-γ plays an important role in controlling both the liver and the blood stage of malaria. However, it can also aggravate malaria infections (38). For example, IFN-γ has been shown to exacerbate the pathology in animal models of cerebral malaria (38, 55). IFN-γ synergizes with lymphotoxin-*α* and TNF to induce the expression of adhesion molecules (E-selectin and ICAM-1) (56). In line herewith, our RNA-Seq analysis in lung endothelial cells showed that IFN-γ induces transcription of vascular cell adhesion protein 1 (VCAM-1) and intercellular adhesion molecule 1 (ICAM-1). Moreover, IFN-γ produced by CD4^+^ T cells upregulates CXCL9 (MIG) and CXCL10 (IP-10) in endothelial cells and induces CD8^+^ T cell recruitment into the brain (39, 57). IFN-γ KO mice are protected from cerebral malaria and show lower leukocyte infiltration in the brain (58).

The involvement of IFN-γ in GC resistance has extensively been studied (59, 60). In the present study, we show that lung endothelial cells remain GC sensitive upon challenge with IFN-γ. However, combined treatment with IFN-γ and *Pb*NK65 extract impairs GC-mediated transcriptional inhibition of proinflammatory cytokines. IFN-γ has been shown to induce GC resistance in other disease models in combination with bacterial products or cytokines. For example, cooperative signaling between IFN-*γ* and LPS induces IL-27 in mouse macrophages and inhibits GR nuclear translocation (59). In airway smooth muscle cells, treatment with IFN-γ and TNF causes GC resistance. Short-term stimulation with IFN-γ and TNF impairs GR binding to DNA and GRE-dependent transcription *via* upregulation of GR-β, whereas long-term treatment depletes GRIP-1 from the GR transcriptional regulatory complexes (60–62). In contrast, Goleva et al. showed that IFN-γ reverses GC resistance induced in T cells by long-term treatment with IL-2 and IL-4 (50).

Various mechanisms leading to GC resistance have been proposed. We show here that treatment with IFN-γ and *Pb*NK65 extract, in the presence or absence of GCs, affects neither GR levels nor GR S211 or S226 phosphorylation in lung endothelial cells. GR S211 phosphorylation is associated with enhanced GR activity, whereas GR S226 phosphorylation exerts inhibitory effects on GR (48, 49, 63). In contrast to our findings, several proinflammatory cytokines have been shown to reduce GR levels or GR S211 phosphorylation. For example, IL-2 and IL-4 impair GR S211 phosphorylation in T cells (50). TNF downregulates the levels of hepatic GR *in vivo*, whereas TGF-β exposure (before challenge with IL-1*α*) reduces GR levels in A549 cells (64, 65). However, TGF-β induced by respiratory syncytial virus fails to downregulate GR levels in human airway epithelial cells (66). Proteasomal degradation of GR reduces its levels and was proposed to cause GC resistance in endothelial cells (32, 33).

Impaired GR nuclear translocation represents another way to mediate GC resistance in various models. In B cells, treatment with IL-4 and IL-15 inhibits GR translocation (31). Similarly, IL-4 and IL-2 impair GR translocation in T cells (50). Superantigens block GR translocation in PBMCs (67). In contrast, GR translocation remains functional in GC resistant HUVECs and lack of response to GCs is associated with defects downstream of GR translocation (68). This is in line with our data, since GR still translocated in the resistant condition with IFN-γ and *Pb*NK65 extract. Moreover, IFN-γ and *Pb*NK65 extract did not affect mRNA levels of FKBP51 (Figure S1 in Supplementary Material), which sequesters GR in the cytoplasm and has been implicated in GC resistance (69, 70).

Since STAT1 signaling mediates transcription of a significant subset of IFN-γ-induced genes, we investigated the effects of GCs on STAT1 activation. In our model, GCs failed to affect STAT1 expression and phosphorylation. Conversely, GCs suppress STAT1 phosphorylation *via* SOCS1 induction in macrophages activated with TLR-ligands (71). Another study showed that GCs inhibit STAT1 expression after long-term incubation in PBMCs treated with IFN-γ but fail to affect its protein stability (72). In macrophages challenged with type I IFN, GCs antagonize STAT1-STAT2-IRF9 (ISGF3) transcriptional complex *via* depletion of GRIP1/TIF2 (used by ISGF3 as coactivator) (73). GRIP1/TIF2 mediates anti-inflammatory actions of GCs in macrophages *via* inhibition of cytokine genes (74). Furthermore, conditional deletion of GRIP1 in obese mice results in macrophage infiltration and inflammation in the liver (75).

Here, we also studied the effect of dexamethasone on the MAPK signaling pathway—a known target of the anti-inflammatory action of GCs. We found that JNK and p38 play an important role in the induction of proinflammatory cytokines in lung endothelial cells upon stimulation with IFN-γ or IFN-γ and *Pb*NK65 extract. Dexamethasone inhibited JNK and p38 phosphorylation upon challenge with IFN-γ. However, this inhibitory effect was lost when cells were stimulated with IFN-γ and *Pb*NK65 extract. GCs have been shown to block MAPK phosphorylation in various experimental models. For example, GCs inhibit JNK *via* protein–protein interaction in HeLa cells. GR interacts with JNK *via* a hormone-regulated JNK docking site in the GR ligand-binding domain and induces disassembly of JNK from mitogen-activated protein kinase kinase 7 (MKK7) (54). In macrophages challenged with LPS, dexamethasone inhibits p38 but neither ERK nor JNK (53). However, dexamethasone blocks JNK phosphorylation in HUVECs stimulated with TNF (76). GCs also inhibit JNK, p38 and ERK phosphorylation in human lung endothelial cells challenged with TNF, IL-1β, and H_2_O_2_ (77).

Numerous studies confirm a highly complex interaction between GR and MAPK signaling. GR phosphorylation by p38 mediates beneficial effects of GCs. For example, GC-induced apoptosis in lymphoid cells requires S211 phosphorylation of GR by p38 (78). Similarly, p38 activation by LPS in a model of acute lung injury enhances anti-inflammatory actions of GR (25). In contrast, JNK negatively regulates the activities of GR by S226 phosphorylation, resulting in enhanced nuclear export and termination of GR signaling (48). MAPK and GR interaction might also result in GC resistance. In airway smooth muscle cells, p38 phosphorylates GR at S203 acting as a negative regulator GR transcriptional activity (79). Inhibition of JNK enhances GR binding to GREs in mouse hippocampal cells (80). Moreover, JNK activation by cholesterol impairs GR-mediated transactivation (81).

MKP-1 (DUSP1) is an important mediator of GC inhibitory actions on MAPK signaling (82, 83). MKP-1 (DUSP1) inhibits p38 in macrophages stimulated with TNF and also blocks JNK and p38 in macrophages challenged with LPS (53, 84). Furthermore, in macrophages from patients with severe asthma, the activity of p38 was increased while expression of MKP-1 (DUSP1) was reduced (29). MKP-1 (DUSP1) also blocks p38 in endothelial cells (76). We observed increased mRNA levels of MKP-1 (DUSP1) after stimulation with IFN-γ and *Pb*NK65 compared to IFN-γ alone in the presence of dexamethasone. Moreover, we evaluated the expression of GILZ upon challenge with IFN-γ and *Pb*NK65 and dexamethasone but we found no difference when compared with IFN-γ alone. GILZ has been suggested to induce MKP-1 (DUSP1) expression. However, silencing of GILZ in HUVECs failed to alter the proinflammatory response to TNF (85, 86).

In conclusion, we here show that *Pb*NK65 extract in combination with IFN-γ impairs GC-mediated transcriptional inhibition of inflammatory chemokines in murine lung endothelial cells. In contrast, lung endothelial cells remain GC sensitive when challenged with IFN-γ alone. GCs block activation of JNK and p38 upon challenge with IFN-γ. However, *Pb*NK65 extract interferes with the inhibitory actions of GCs on p38 and JNK. This work offers an interesting explanation of our previous observation in the preclinical model of MA-ARDS, namely that GCs could only block the pulmonary inflammation at extremely high dosages and were even then not able to downregulate the expression of several inflammatory genes in the lungs (9). Our data provide a unique view on the regulation of IFN-γ-induced inflammation and identify *P. berghei* NK65 as a novel inducer of GC resistance in lung endothelial cells.

## Ethics Statement

All animal experiments were performed in accordance to the regulations as declared in Directive 2010/63/EU from the European Union and and the Belgian Royal Decree of 29 May 2013 and were approved by the Animal Ethics Committee from the KU Leuven (project number P163-2014, License LA1210186, Belgium).

## Author Contributions

KZ, LDC, SK, KVDM, JT, and AS performed the experiments. KZ, LDC, AS, KDB, and PVDS analyzed and interpreted the data. PVDS, KDB, KZ, GO, and JBDS conceived the study and designed the experiments. The manuscript was written by KZ and further edited by PVDS, KDB, and GO. All authors critically read and approved the manuscript.

## Conflict of Interest Statement

The authors declare that the research was conducted in the absence of any commercial or financial relationships that could be construed as a potential conflict of interest.
