# Supplementary material for: Plasmodium berghei NK65 in Combination with IFN-γ Induces Endothelial Glucocorticoid Resistance via Sustained Activation of p38 and JNK
Source: Front Immunol. 2017 Sep 28;8:1199. doi: 10.3389/fimmu.2017.01199 (PMC5625030; doi:10.3389/fimmu.2017.01199)
Supplement: Supplementary file 1 [file supplementary_material.pdf]

# *Supplementary Material*: Plasmodium berghei NK65 in combination with IFN-$\gamma$ induces endothelial glucocorticoid resistance via sustained activation of p38 and JNK

**Karolina A Zielińska, Lode de Cauwer, Sofie Knoops, Kristof Van der Molen, Alexander Sneyers, Jonathan Thommis, J. Brian De Souza, Ghislain Opdenakker, Karolien De Bosscher and Philippe E. Van den Steen***

*Correspondence:
Author Name: Prof. Philippe E Van den Steen, Herestraat 49 box 1044, 3000 Leuven, Belgium
philippe.vandensteen@kuleuven.be

## 1 SUPPLEMENTARY TABLES AND FIGURES

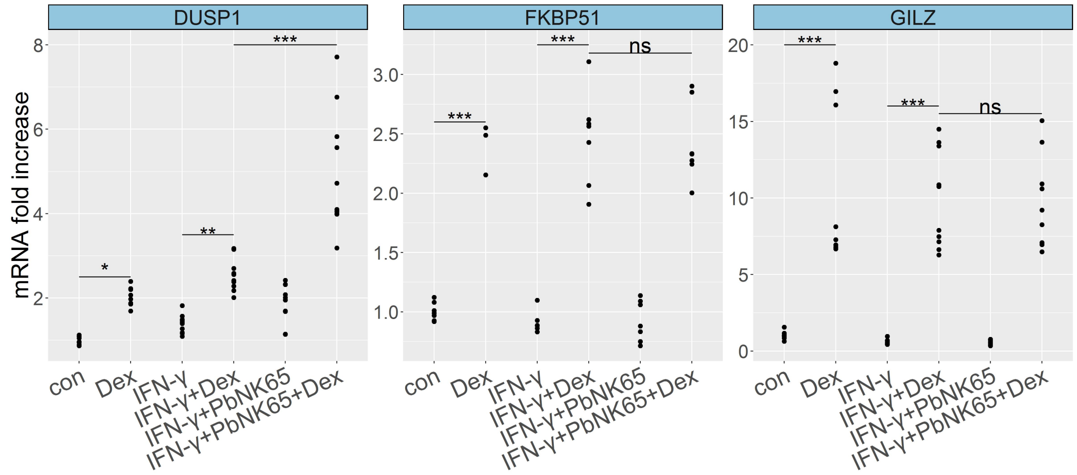


**Figure S1.** Dexamethasone induces DUSP-1 (MKP1), FKBP51 and GILZ in IFN-$\gamma$ or IFN-$\gamma$ and *Pb*NK65-treated lung endothelial cells. L2 MVECs were stimulated with vehicle (con), IFN-$\gamma$ (20 ng/mL) or IFN-$\gamma$ and *Pb*NK65 extract (*Pb*NK65, $10^7$ infected RBCs/mL) in the presence or absence of dexamethasone (Dex, 100 nM) for 24 hours. DUSP-1 (MKP1), FKBP51 and GILZ expression was analyzed by qRT-PCR. Statistical significance was evaluated using ANOVA (* $p$ <0.05, ** $p$ <0.01, *** $p$ <0.001). Data represent combined results from at least 3 independent experiments.

| Rank | Motif / Name | p-value | q-value | % of target sequences with motif |
|---|---|---|---|---|
| 1 | 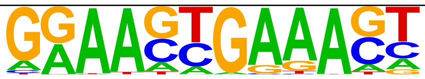 IRF8(IRF)/BMDM-IRF8-ChIP-Seq(GSE77884) | 1e−13 | < 1e−4 | 40.26% |
| 2 | 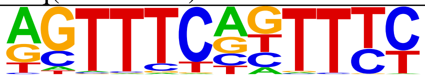 IRF3(IRF)/BMDM-Irf3-ChIP-Seq(GSE67343) | 1e−13 | < 1e−4 | 36.36% |
| 3 | 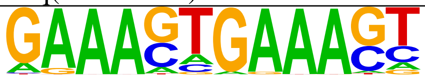 IRF1(IRF)/PBMC-IRF1-ChIP-Seq(GSE43036) | 1e−11 | < 1e−4 | 25.97% |
| 4 | 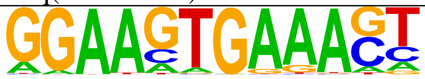 PU.1:IRF8(ETS:IRF)/pDC-Irf8-ChIP-Seq(GSE66899) | 1e−9 | < 1e−4 | 27.27% |
| 5 | 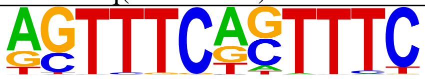 ISRE(IRF)/ThioMac-LPS-Expression(GSE23622) | 1e−8 | < 1e−4 | 15.58% |
| 6 | 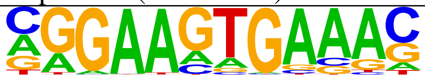 PU.1-IRF(ETS:IRF)/Bcell-PU.1-ChIP-Seq(GSE21512) | 1e−7 | < 1e−4 | 63.64% |
| 7 | 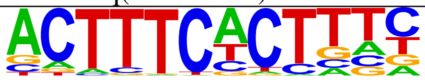 PRDM1(Zf)/Hela-PRDM1-ChIP-Seq(GSE31477) | 1e−7 | < 1e−4 | 35.06% |
| 8 | 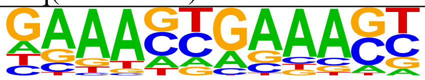 IRF2(IRF)/Erythroblas-IRF2-ChIP-Seq(GSE36985) | 1e−4 | 0.0013 | 14.29% |
| 9 | 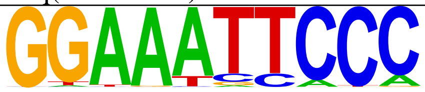 NFkB-p65-Rel(RHD)/ThioMac-LPS-Expression(GSE23622) | 1e−4 | 0.0014 | 10.39% |
| 10 | 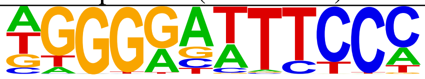 NFkB-p65(RHD)/GM12787-p65-ChIP-Seq(GSE19485) | 1e−3 | 0.004 | 28.57% |

Table S1Top 10 enriched transcription factor-binding motifs in -400 to +100 bp in genes induced by IFN-$\gamma$ at least 2 fold and repressed by dexamethasone at least by 50%.

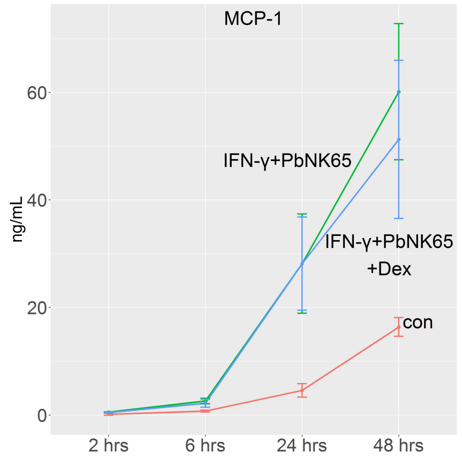


**(2a)**

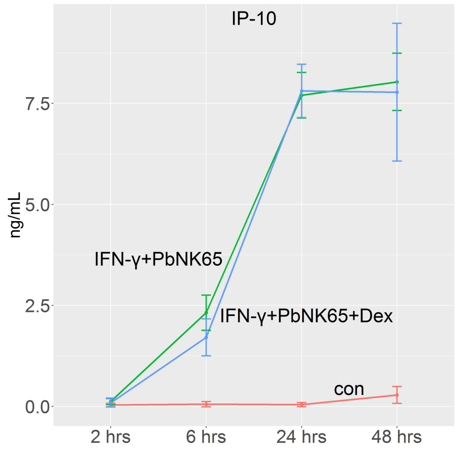


**(2b)**

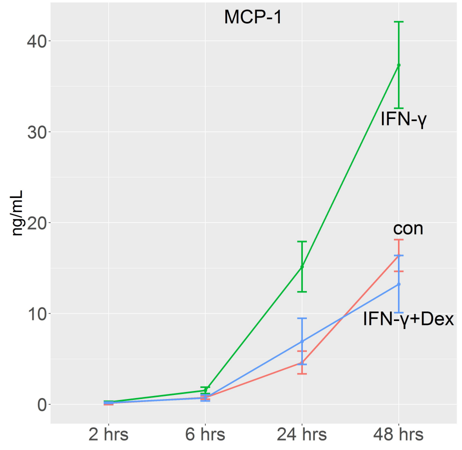


**(2c)**

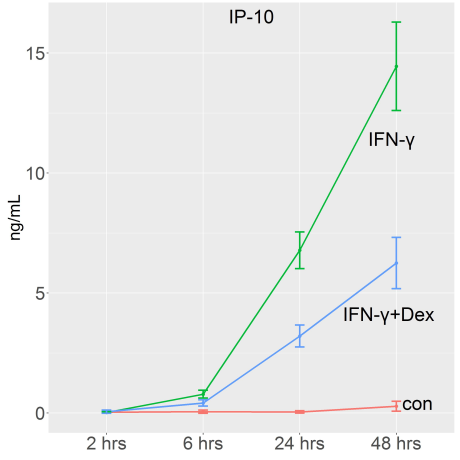


**(2d)**

**Figure S2.** *Pb*NK65 extract in combination with IFN-$\gamma$ induces GC resistance after 6 or 24 hours. L2 MVECs were stimulated with vehicle (con, red), IFN-$\gamma$ (20 ng/mL, green) and IFN-$\gamma$ and *Pb*NK65 extract (*Pb*NK65, $10^7$ infected RBCs/mL, blue) in the presence or absence of dexamethasone (Dex, 100 nM). MCP-1 (CCL2) and IP-10 (CXCL10) levels in culture supernatant were analyzed by ELISA. Data are presented as mean of 2 independent experiments +/- SD (n=4).

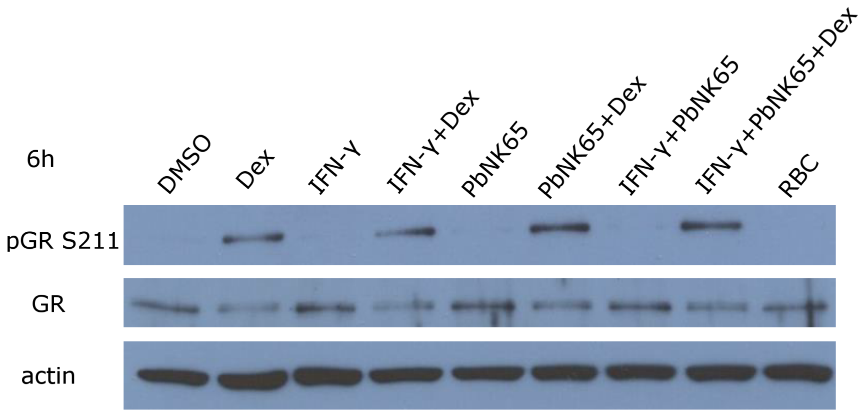


**Figure S3.** IFN-$\gamma$ and *Pb*NK65 extract do not interfere with either GR expression or GR phosphorylation. Western blot analysis of lysates of L2 MVECs stimulated for 6 hours with vehicle (DMSO), red blood cells extract (RBC, $10^7$ RBCs/mL), IFN-$\gamma$ (20 ng/mL), *Pb*NK65 extract ($10^7$ infected RBCs/mL), IFN-$\gamma$ and *Pb*NK65 extract in the presence or absence of dexamethasone (Dex, 100 nM).

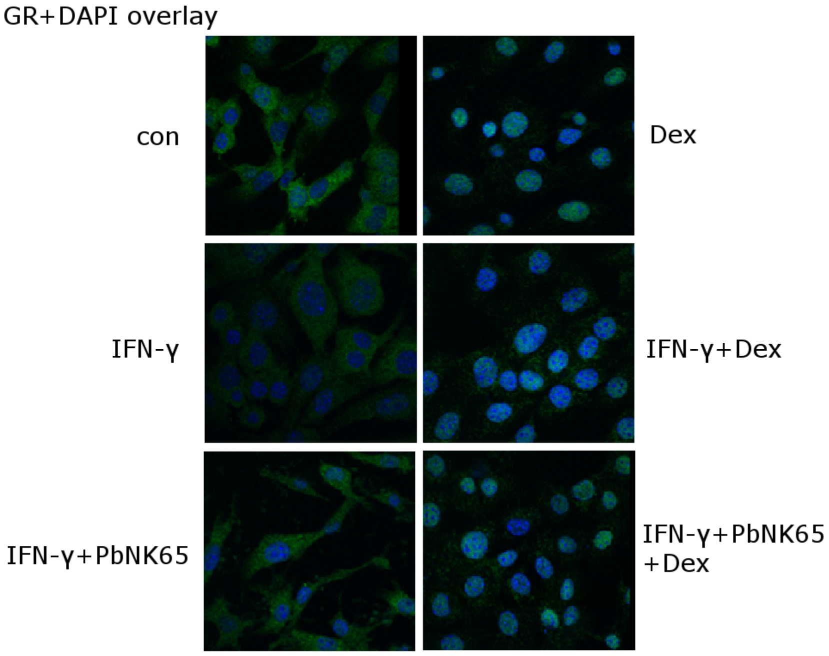


**(4a)**

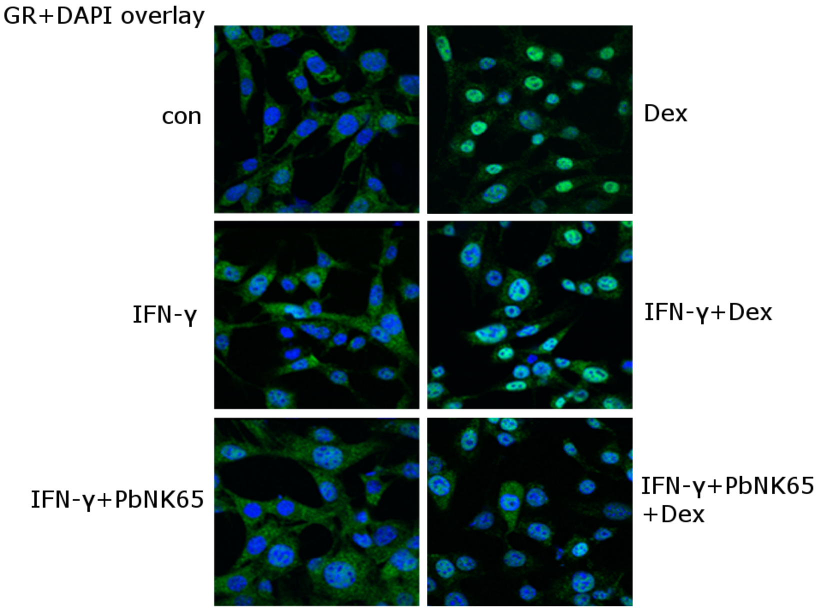


**(4b)**

**Figure S4.** IFN-$\gamma$ and *Pb*NK65 do not interfere with GR translocation after 24 or 2 hours. L2 MVEC cells were stimulated with solvent (con), IFN-$\gamma$ (20ng/mL) or IFN-$\gamma$ and *Pb*NK65 extract ($10^7$ infected RBCs/mL) in the presence or absence of dexamethasone (Dex, 100nM) for 24 hours (a) or for 2 hours and treated for 1 hour with dexamethasone (b). Endogenous GR was visualized (green) through indirect immunofluorescence using anti-GR Ab. DAPI staining (blue) indicates the nuclei of the cells.

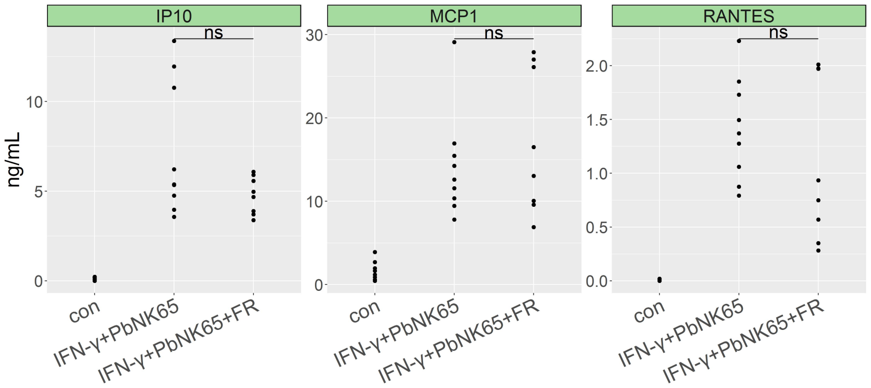


**Figure S5.** ERK inhibitor fails to impair pro-inflammatory cytokines expression in lung endothelial cells stimulated with IFN-$\gamma$ and *Pb*NK65 extract. L2 MVECs were stimulated with IFN-$\gamma$ (20 ng/mL) and *Pb*NK65 extract (*Pb*NK65, $10^7$ infected RBCs/mL) in the presence of ERK inhibitor (FR180204, 10 $\mu$M) for 24 hours. Protein levels of CXCL10 (IP-10), CCL2 (MCP-1) and CCL5 (RANTES) were determined. Statistical significance was evaluated using Mann-Whitney test. Data show combined results from 3 independent experiments.

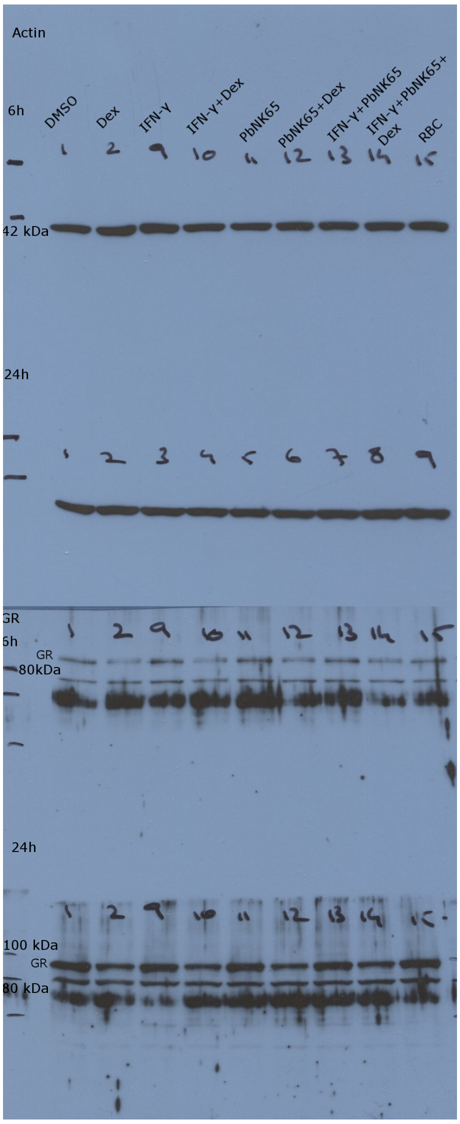


(6a)

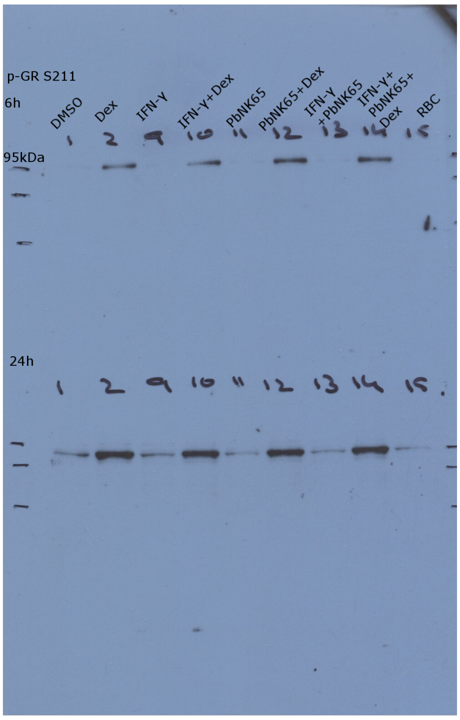


(6b)

**Figure S6.** Whole Western blots of GR (panel a) and pGR S211 (panel b) from L2 MVECs stimulated for 6 or 24 hours with vehicle (DMSO), IFN-$\gamma$ (20 ng/mL), IFN-$\gamma$ and *Pb*NK65 extract (*Pb*NK65, $10^7$ infected RBCs/mL) and red blood cells extract (RBC, $10^7$ RBCs/mL) in the presence or absence of dexamethasone (Dex, 100 nM).

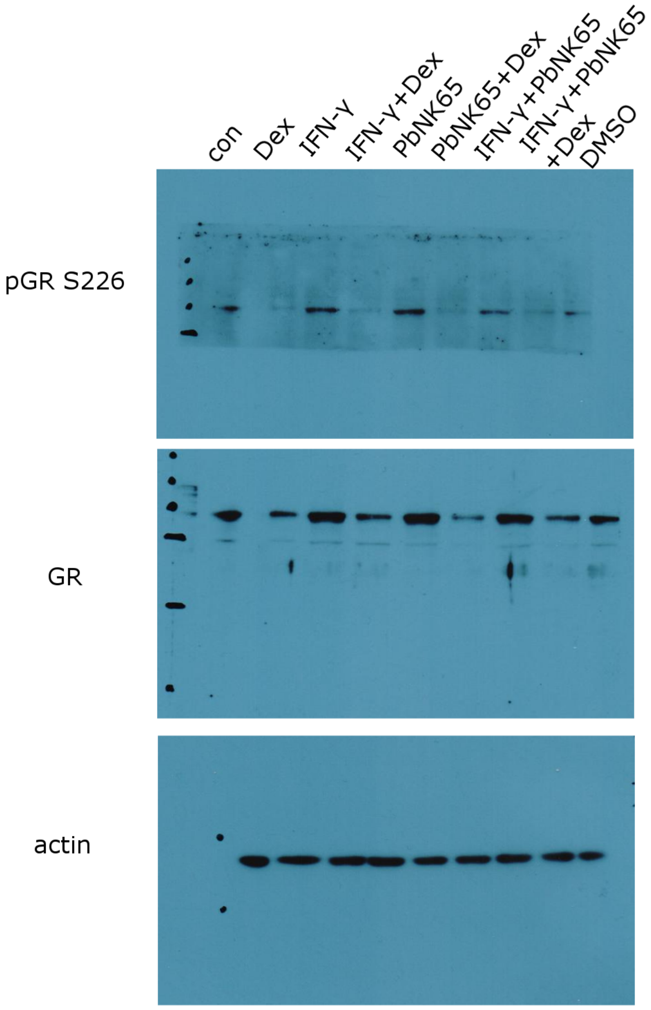


**Figure S7.** Whole p-GR (S226) and GR Western blots for L2 MVECs stimulated for 24 hours with vehicle (DMSO), IFN-$\gamma$ (20 ng/mL), IFN-$\gamma$ and *Pb*NK65 extract (*Pb*NK65, $10^7$ infected RBCs/mL) in the presence or absence of dexamethasone (Dex, 100 nM).

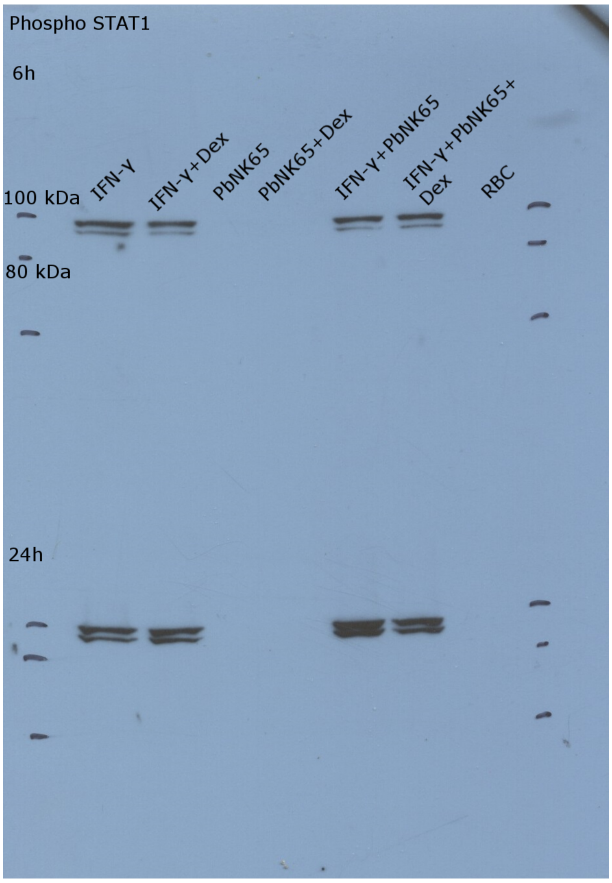


**(8a)**

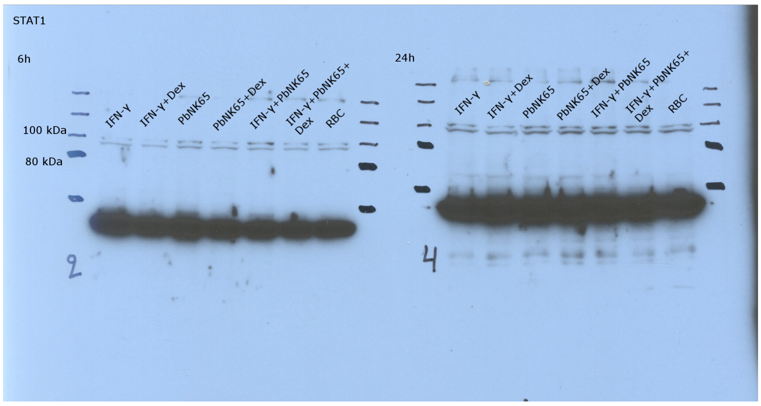


**(8b)**

**Figure S8.** Whole p-STAT1 (panel a) and STAT1 (panel b) Western blots for L2 MVECs stimulated for 6 or 24 hours with vehicle (DMSO), IFN-$\gamma$ (20 ng/mL), IFN-$\gamma$ and *Pb*NK65 extract (*Pb*NK65, $10^7$ infected RBCs/mL) and red blood cells extract (RBC, $10^7$ RBCs/mL) in the presence or absence of dexamethasone (Dex, 100 nM).

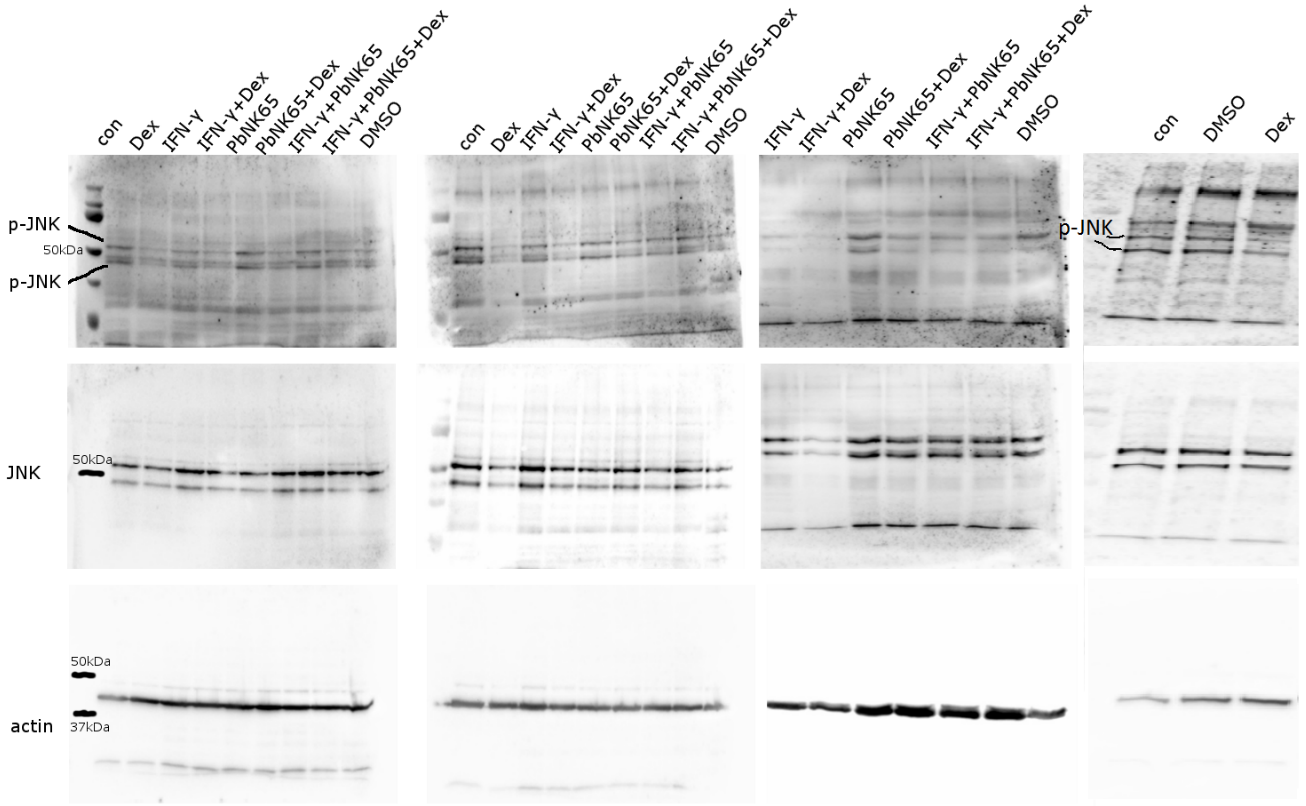


**Figure S9.** Whole p-JNK and JNK Western blots for L2 MVECs stimulated for 24 hours with vehicle (con), IFN-$\gamma$ (20 ng/mL), IFN-$\gamma$ and *Pb*NK65 extract (*Pb*NK65, $10^7$ infected RBCs/mL) in the presence or absence of dexamethasone (Dex, 100 nM). Data show 3 independent experiments and extra control analyses.

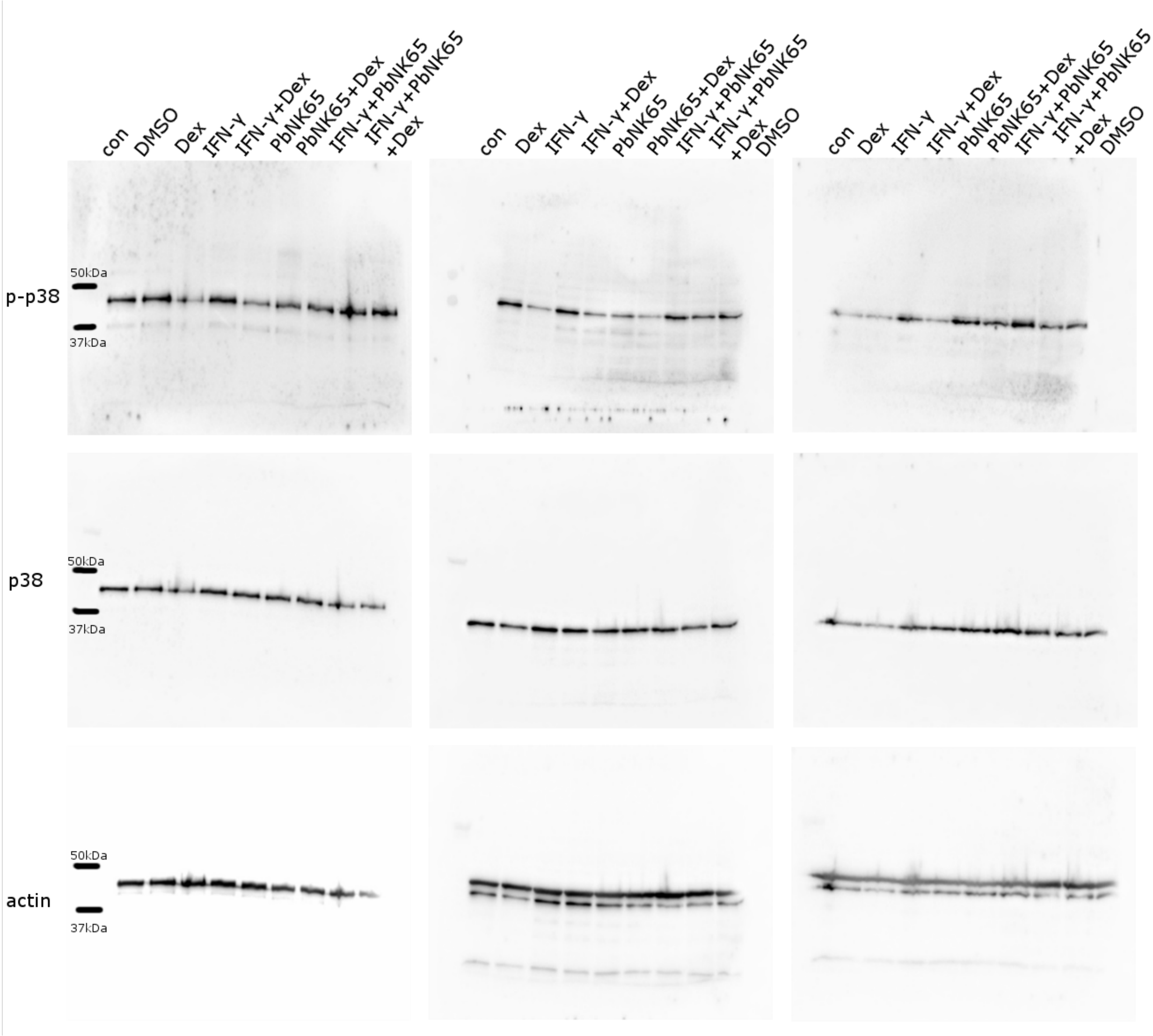


**Figure S10.** Whole p-p38 and p38 Western blots for L2 MVECs stimulated for 24 hours with vehicle (con), IFN-$\gamma$ (20 ng/mL), IFN-$\gamma$ and *Pb*NK65 extract (*Pb*NK65, $10^7$ infected RBCs/mL) in the presence or absence of dexamethasone (Dex, 100 nM). Data show 3 independent experiments.
