# Supplementary figures and images for: Plasmodium berghei NK65 in Combination with IFN-γ Induces Endothelial Glucocorticoid Resistance via Sustained Activation of p38 and JNK
Source: Front Immunol. 2017 Sep 28;8:1199. doi: 10.3389/fimmu.2017.01199 (PMC5625030; doi:10.3389/fimmu.2017.01199)

Heatmap of relative log-transformed values for  
top 100 highly variable genes across samples

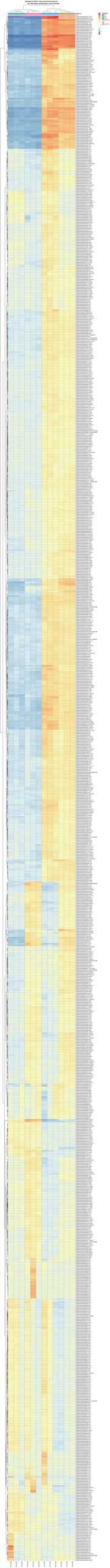

Supplement: Supplementary file 2 [file supplementary_file_1.pdf]
